# Recent Advances in the Development of Polymyxin Antibiotics: 2010–2023

**DOI:** 10.1021/acsinfecdis.3c00630

**Published:** 2024-03-12

**Authors:** Cornelis J. Slingerland, Nathaniel I. Martin

**Affiliations:** † Biological Chemistry Group, Institute of Biology Leiden, Leiden University, Sylviusweg 72, 2333 BE Leiden, The Netherlands

## Abstract

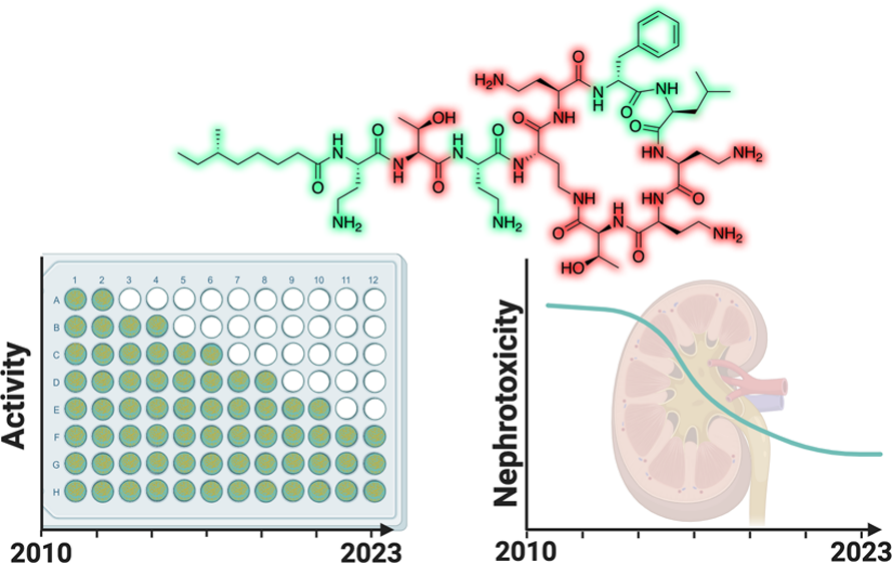

The polymyxins are nonribosomal lipopeptides produced by *Paenibacillus polymyxa* and are potent antibiotics with activity specifically directed against Gram-negative bacteria. While the clinical use of polymyxins has historically been limited due to their toxicity, their use is on the rise given the lack of alternative treatment options for infections due to multidrug resistant Gram-negative pathogens. The Gram-negative specificity of the polymyxins is due to their ability to target lipid A, the membrane embedded LPS anchor that decorates the cell surface of Gram-negative bacteria. Notably, the mechanisms responsible for polymyxin toxicity, and in particular their nephrotoxicity, are only partially understood with most insights coming from studies carried out in the past decade. In parallel, many synthetic and semisynthetic polymyxin analogues have been developed in recent years in an attempt to mitigate the nephrotoxicity of the natural products. Despite these efforts, to date, no polymyxin analogues have gained clinical approval. This may soon change, however, as at the moment there are three novel polymyxin analogues in clinical trials. In this context, this review provides an update of the most recent insights with regard to the structure–activity relationships and nephrotoxicity of new polymyxin variants reported since 2010. We also discuss advances in the synthetic methods used to generate new polymyxin analogues, both via total synthesis and semisynthesis.

## Introduction: Polymyxins, Classic but Crucial Antibiotics

Antibiotics are a cornerstone of modern healthcare but their viability is threatened significantly by the emergence of antimicrobial resistance (AMR).^1^ This problem is particularly noteworthy in hospital settings where the intense use of antibiotics is increasingly giving rise to nosocomial infections. In 2019, 1.27 million people died globally as a direct consequence of AMR, while an additional 3.68 million deaths were associated with AMR.^2^ Among the pathogens contributing to these mortality rates are antibiotic resistant isolates of the Gram-negative species *K. pneumoniae*, *A. baumannii*, *P. aeruginosa*, and *E. coli*.^2^ While integrated health approaches such as monitoring and stewardship can mitigate the infectious potential of such drug-resistant pathogens, it is clear that new antibiotics are required to treat the infections they cause. The development of entirely new antibiotics operating via unexploited mechanisms requires major investment and carries significant risk for drug makers which has led to their reduced enthusiasm for such campaigns in recent decades.^3,4^ As an alternative, the improvement of clinically used natural product antibiotics has, and continues to be, a proven and fruitful strategy in antibiotic discovery that is likely to continue to be of value in addressing the increasing antibiotics crisis.^5,6^

The polymyxins are a class of natural product lipopeptide antibiotics that specifically prevent the growth of Gram-negative bacteria. They do so by targeting a unique biomolecular target found only on the outside of Gram-negative cells: lipid A, the membrane associated anchor of lipopolysaccharide (LPS).^7−9^ LPS decorates most Gram-negative bacteria on the surface of their outer membrane (OM) and is responsible for many of the systemic toxic and inflammatory effects associated with infections caused by Gram-negative bacteria.^10,11^ The polymyxins were discovered and brought to the clinic in the so-called golden era of antibiotic discovery (1940–1960).^12,13^ Many antibiotics discovered in this era, including the polymyxins, subsequently fell out of favor as newer generations of antibiotics with improved efficacy and tolerability profiles were developed. However, with the advent and spread of multidrug resistant (MDR) bacteria, classic antibiotics like the polymyxins face renewed interest. While the toxicities of the polymyxins present a clear drawback to their clinical application, their potent activity against numerous Gram-negative bacteria continues to make them an important last-resort option. Many medicinal chemistry campaigns have been conducted in an attempt to reduce the toxicity of the polymyxins while preserving their potent antibacterial activity. Also of note are recent studies with the octapeptins, a family of lipopeptide antibiotics isolated from *B. circulans* with structural similarity to polymyxins.^14^ However, compared to the polymyxins, significantly fewer medicinal chemistry studies have been reported in pursuit of octapeptin analogues with improved properties and as such they are not covered here. In this review we focus on the medicinal chemistry efforts associated with accessing next-generation polymyxins reported since 2010. For an account of the many advances made prior to this period we direct the reader to excellent reviews published previously.^15−17^

## Polymyxins: Structural Features, Toxicity, and Mechanisms of Action

### Polymyxins from Natural Sources

The first polymyxin to be discovered was polymyxin A (initially named aerosporin), isolated from fermentations of *Bacillus aerosporus* (later renamed *Paenibacillus polymyxa*) in the 1940s.^18−20^ In the years that followed a number of structurally similar lipopeptide antibiotics were also identified by culturing approaches resulting in the recognition of the broader polymyxin family as summarized in previous comprehensive reviews.^15,21−23^ Among all known polymyxins, the only clinically used agents are polymyxin B and polymyxin E, with the latter also commonly referred to as colistin.^24^ More recently, genome mining has gained popularity as a means of prospecting for natural products, which led to the identification of macolacin, a polymyxin produced by *Paenibacillus xylanexedens*.^25^ Interestingly, the amino acid composition of macolacin is identical with that of polymyxin F which was first reported in the 1970s but for which a full structure was not determined.^22,26^

**Figure 1:**
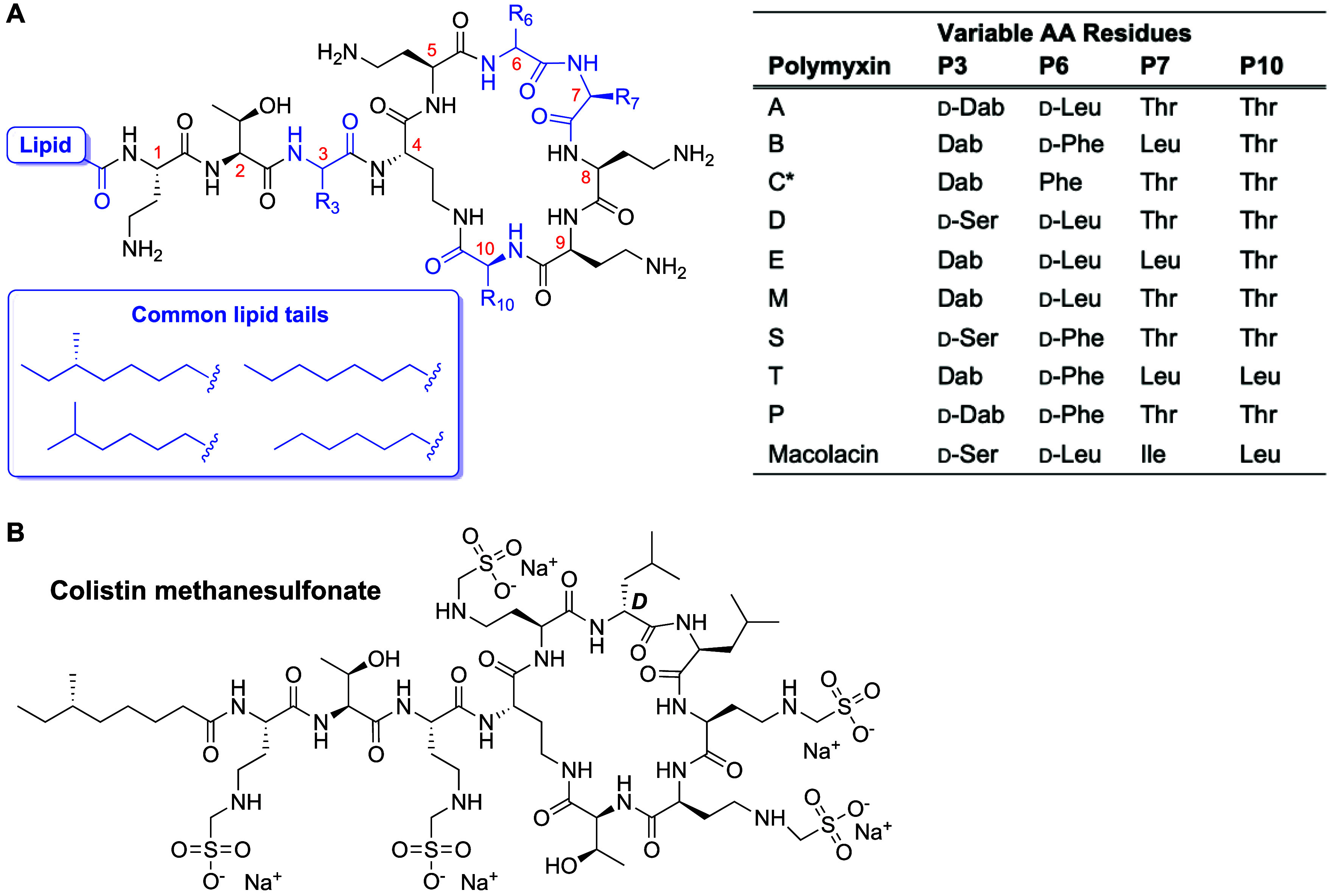
(A) General structure of the naturally occurring polymyxins, with residue numbering indicated and variable features highlighted in blue. *For polymyxin C, the stereochemistry of the constituent amino acids has not been reported. (B) Structure of the clinically used colistin methanesulfonate, a prodrug form of polymyxin E developed in an attempt to reduce the toxicity associated with polymyxins.

All polymyxins are composed of a peptide macrocycle consisting of seven residues connected to an exocyclic tripeptide that is *N*-acetylated with a lipid tail (Figure 1A). In general, amino acid numbering in the polymyxin class follows the formalism indicated in Figure 1A. Throughout the text of this review when reference is made to a specific residue position it will be indicated by “P*x*”, with “*x*” denoting the indicated position. Common to all polymyxin variants is the presence of the nonproteinogenic residue diaminobutyric acid (Dab) at positions 1, 4, 5, 8, and 9. The side chain of Dab4 is connected with the polymyxin C-terminal carboxyl group of Thr10 to create the heptapeptide macrocycle. The other four Dab residues are protonated at physiological pH giving the polymyxins their polycationic character. With the exception of these five Dab residues and Thr2, the other amino acids in the polymyxin scaffold are found to vary among the naturally occurring family members. The P6 position is typically filled with a nonpolar d-amino acid (generally d-Leu or d-Phe). Interestingly, the configuration at P3 is variable as either l- or d-stereochemistry can be encountered at this position among the naturally occurring polymyxins. The *N*-terminal acyl tails found in all natural polymyxins are most commonly derived from the branched (*S*)-6-methyl octanoic or 6-methyl heptanoic acids, as well as from the linear octanoic or heptanoic acids (Figure 1A).^15,21−23^ Typically, fermentative production yields an ensemble of polymyxin isomers usually owing to the incorporation of these different lipid tails.^27^ As also illustrated in Figure 1A, diversity in the peptide sequence is observed, either as a result of the presence of a specific Biosynthetic Gene Cluster (BGC) in the producing host, or as a consequence of substrate flexibility of the enzymes involved in Nonribosomal Peptide Synthesis (NRPS).^28^

It is also well documented that while truncated polymyxin derivatives, most notably polymyxin B nonapeptide (PMBN), are much less active than the parent compound, they maintain the ability to disrupt the Gram-negative OM.^29−33^ In this way PMBN and related polymyxin derivatives have been widely explored as synergists capable of enhancing the activity of antibiotics that otherwise cannot traverse the OM. It is also noteworthy that PMBN is significantly less nephrotoxic than the parent compound, providing some insights into the structural features that contribute to the undesired effects associated with the polymyxins.^34,35^ Given that the focus of this review is on polymyxins with direct antibiotic activity and not on polymyxin-based synergists, we direct the reader to another recently published review that provides an up to date view of OM-disrupting synergists.^32^

### Polymyxin Toxicity

The polymyxins are associated with a range of toxicity issues, most notably nephrotoxicity along with some reports of neurotoxicity.^36−42^ From an early stage in the their clinical history, serious side effects were noted upon administering polymyxins.^43^ Upon systemic administration, polymyxins accumulate primarily in the proximal tubule (PT) cells of the renal cortex, as visualized by exposing kidney tissue from polymyxin treated mice to polymyxin specific IgM antibodies.^44^ Polymyxins have also been shown to accumulate in rat PT cells (NRK-52E) and human PT cells (HK-2), based on the detection of a dansyl-labeled polymyxin analogue. Using this approach, it was determined that the concentration of polymyxin found inside PT cells was 1000-fold higher than the extracellular concentration.^45^

Mechanistically, uptake of polymyxins by the kidney occurs primarily via the apical membrane of proximal tubule epithelial cells. The uptake is saturable, suggesting that a transporter is involved.^46^ Several studies have shown that megalin (also called LRP2), an endocytic receptor^47^ involved in the uptake of polybasic drugs,^48^ plays a role in the uptake of polymyxins into renal cells.^46,49,50^ Two other apical receptors have also been identified as polymyxin uptake receptors: the carnitine/organic cation transporter 2 (OCTN2) and oligopeptide transporter 2 (PEPT2).^51−53^ Recent work by Li and co-workers also suggests that the inwardly rectifying potassium channels Kir4.2 and Kir5.1 may function as transmembrane proteins that mediate the uptake of polymyxins in HK-2 cells.^54^ Notably, the interaction of these channels with polymyxins is proposed to not only allow for polymyxin uptake, but also for inward potassium flux, changes of membrane potential, and cellular depolarization.^54,55^ Megalin ligands like endogenous cytochrome c and cilastatin (used clinically to inhibit renal dehydropeptidase I) inhibit polymyxin uptake into renal cells. Colchicine, a microtubule polymerization inhibitor required for physiological megalin action, does the same.^49,56^ For OCTN2, a protective effect is seen upon supplementation with carnitine, effectively reducing the cytotoxic effects of polymyxins.^51^ For Kir4.2 and Kir5.1, the universal Kir inhibitor BaCl_2_ and the specific Kir4.2 inhibitor VUF0134992^57^ were found to provide protection against polymyxin induced toxicity on HK-2 cells.^54^ The relative contribution of each of these uptake receptors for polymyxin toxicity remains to be elucidated.

Polymyxin localization in the nuclear region and throughout the cytoplasm has been shown both in rat (NRK-52E) and human (HK-2) PT derived cell lines.^45^ Association with the ER, but not with lysosomes, has also been shown by means of a similarly dansyl-labeled polymyxin analogue.^58^ This indicates a pathway wherein endocytosis of polymyxins is followed by endosomal escape, a process observed for many biological substances.^59^ Intracellular effects of polymyxin accumulation include DNA instability, as observed by double stranded breaks in HK-2 cells treated with polymyxin B.^60^ This finding is in line with earlier observations that polymyxins can bind directly to dsDNA strands.^61^ Increased levels of reactive oxygen species (ROS) have also been found in cells incubated with polymyxins.^62,63^ ROS are involved in the induction of apoptosis via several pathways.^64^ Studies in mice have shown apoptosis in renal cells upon polymyxin treatment.^62,65^ Freshly isolated mouse mitochondria also show a loss of Ca^2+^ and membrane potential upon incubation with colistin.^53^ As a consequence of the involvement of ROS and oxidative stress, many compounds have been screened for their potential to lower oxidative stress upon polymyxin treatment.^66^ These include antibiotics (minocycline),^67^ immunosuppressants (rapamycin),^68^ dietary flavonoids (rutin)^69^ and curcumin,^70,71^ a compound with alleged antioxidant properties.^72^

While it may be possible to attenuate polymyxin toxicity by coadministration of agents capable of reducing these effects, a more direct strategy would be to address the toxicity of polymyxins themselves thus avoiding the need for combination therapy and its associated drawbacks. In this regard, derivatization of colistin to the methanesulfonate (CMS) adduct (Figure 1B) was an approach developed in the 1960s in an attempt to reduce its systemic toxicity.^73^ In general, CMS is considered to be an inactive prodrug.^74−76^ Upon metabolic conversion to the parent compound *in vivo*, antibacterial activity is restored, making the therapeutic benefit largely dependent on PK properties. Recently, the actual benefit of this approach has been called into question and the therapeutic preference of clinicians for colistin, CMS, or polymyxin B remains an ongoing debate.^77−80^ This review is primarily focused on recent advances in the development of next generation polymyxins that address the toxicity issues associated with their use. Before expanding on this topic, we will also address the mechanistic underpinnings of the specific anti-Gram-negative activity of the polymyxins, including a number of recently reported insights.

### Mechanisms of Action

As is the case for many lipopeptide antibiotics, the mechanism underscoring the antibacterial activity of the polymyxins includes several aspects, many of which have been covered in previous reviews.^15,16,81,82^ In addition, while new mechanisms of resistance to polymyxins have emerged in the past decade, we will not delve into these in detail here and instead direct the reader to recently published high quality reviews that are specifically focused on the topic of polymyxin resistance.^83−86^ A basic understanding of the polymyxin mechanism of action includes an initial electrostatic interaction between the cationic Dab residues of the polymyxin and the anionic phosphate head groups of lipids in the bacterial OM, typically lipid A. This association results in the displacement of Mg^2+^ and Ca^2+^ ions that serve to bridge the phosphate headgroup of adjacent lipid A molecules.^16^ Upon lipid A binding, the polymyxin molecule is then believed to adopt a conformation that separates its polar and nonpolar features. In doing so, the polymyxin’s acyl tail and hydrophobic Leu-d-Phe motif fold toward the lipophilic lipid A tails, while the more polar side chains point toward the sugar and phosphate regions of lipid A.^87,88^

Given that LPS/lipid A is highly abundant in the OM, much attention has been focused on this initial interaction as the driving force behind the activity of the polymyxins. However, a variety of LPS precursors, including lipid A variants, are also found in the inner membrane (IM).^11^ While the amount of LPS in the OM far exceeds its abundance in the IM, recent studies indicate that the presence of LPS in the IM also contributes significantly to polymyxin’s mechanisms of action.^89−91^ Edwards and co-workers recently investigated the impact of colistin on the cytoplasmic membrane of Gram-negative bacteria.^90,92^ Specifically, by using spheroplasts derived from *E. coli* cells, they could assess the effects of colistin on the cytoplasmic membrane isolated from the OM. In addition, studies with polymyxin resistant strains wherein resistance is conferred by *mcr* plasmids encoding phosphoethanolamine transferases that modify the structure of lipid A, have provided new insights into the relative importance of polymyxin interactions with the OM and IM.^90^ Notably, among such resistant strains a significantly larger portion of LPS in the IM was found to be modified by ethanolamine compared to LPS in the OM. When treated with colistin, the OM was still readily permeabilized, but not the IM. This implies that *mcr* positive strains are resistant toward colistin primarily by virtue of ethanolamine modified LPS in their IM, not in their OM.

In keeping with these findings, the Edwards group also showed that treatment with murepavadin, a *P. aeruginosa* specific LptD inhibitor,^93^ inhibited LPS transport to the OM.^90^ Upon coadministration of murepavadin and colistin on *P. aeruginosa*, a synergistic effect was observed. The authors ascribed this effect to the capacity of murepavadin to interfere with the transport of LPS to the OM, leading to an accumulation of LPS in the IM, and in doing so enhancing the IM-specific effect of colistin. Notably, Kahne and co-workers had previously reported that novobiocin analogues that stimulate LptB, one of the proteins involved in LPS transport from the IM to the OM, can also induce more efficient killing by polymyxin.^94^ It has been postulated that increased LPS transport will lead to enhanced LPS synthesis, yielding increased levels both in the IM and OM.^90^ This hypothesis may reconcile the observations of both the Kahne and Edwards groups as it seems that interfering with LPS transport (whether via LptD or LptB) can potentiate the bactericidal action of polymyxins.

### Supramolecular Effects

Recently, Hiller and co-workers published a study describing the interaction of colistin with outer membrane vesicles (OMVs) derived from both polymyxin-susceptible and -resistant strains.^95^ This revealed a clear correlation between the efficacy of the antibiotic and the resulting macromolecular features observed on the OMV surface by use of atomic force microscopy (AFM). Upon adding colistin to OMVs derived from polymyxin-sensitive strains, a clear hexagonal or honeycomb-like pattern was observed by AFM imaging, suggesting an ordered rearrangement of OMV associated molecules. This effect was found to be independent of OM proteins and highly dependent on the presence of divalent cations. When OMVs containing truncated LPS were used, the same hexagonal pattern was observed, in line with our group’s assessment of the activity of polymyxins on strains bearing truncated LPS variants.^96^ Interestingly, a polymyxin analogue (d-Thr at P10) with reduced activity (MIC: 32 μg/mL) elicited a similar hexagonal pattern, while a much less active analogue (azido-Dab at P9, MIC: 128 μg/mL) did not. The authors further note that induction of the hexagonal crystalline pattern is associated with lateral membrane expansion, explaining previously observed protrusions in bacterial membranes upon polymyxin treatment.^91^ In addition to the Hiller group, several others have studied polymyxin-target interactions using AFM. Notably, an increase in cell surface roughness was observed, as well as the formation of micro- and nanoclusters.^91,97^ The higher resolution achieved in the studies performed by Hiller and co-workers likely provides for a more detailed explanation of the phenomena observed in earlier investigations. It is also worth noting that while OMVs provide a very good representation of the OM, they do not allow the study of effects on the IM. In this light, evidence derived from studies using OMVs alone needs to be considered as providing only part of the picture, as is also the case for insights gleaned from studies using only spheroplasts.

### Stereochemical Requirements

The first investigations aimed at assessing the stereochemical underpinnings of the polymyxin mechanism of action employed the enantiomeric form of polymyxin B nonapeptide, *ent*-PMBN, rather than the full-length natural product.^98^ In these studies, the Fridkin group prepared *ent*-PMBN by total synthesis and somewhat surprisingly found that its binding to LPS appeared to be similar to PMBN. These investigations relied on a displacement assay, in which dansyl-PMBN, bound to LPS, was shown to be readily displaced by *ent*-PMBN. In support of this finding, binding studies recently performed in our group using isothermal titration calorimetry (ITC) revealed identical LPS binding patterns for PMBN and *ent*-PMBN.^96^ Interestingly, however, was the observation that *ent*-PMBN does not potentiate the activity of novobiocin against *E. coli*, while PMBN does so strongly. This finding led Fridkin and co-workers to conclude that the association of PMBN or *ent*-PMBN with LPS has relatively low stereochemical requirements, whereas the functional association leading to synergy with novobiocin requires the native stereoisomeric species.^98^

A recent study by the Reymond group also provided insights into the stereochemical requirements for polymyxin activity by means of stereorandomization, wherein racemic amino acids were introduced into the polymyxin sequence.^99^ Somewhat surprisingly, the mixture that resulted from full stereorandomization of polymyxin B (yielding a theoretical mixture of 1024 possible diastereomers), still showed activity on *E. coli* (MIC: 2 μg/mL). Partial stereorandomization, either at positions 1, 3, 4, 7, 9, 10 (64 possible diastereomers) or at positions 2, 3, 4, 9, 10 (32 possible diastereomers) also yielded active mixtures, with the latter achieving an MIC of 0.25 μg/mL on *E. coli* and 2 μg/mL on *A. baumannii*. It should be noted that the relative amount of each possible diastereomer within these mixtures was not quantified, raising the possibility that some are overrepresented, influencing the activity of the mixture. Interestingly, the mixtures of diastereomers were found to be less efficient in LPS binding, as judged by the level of TNF-α expression in RAW264.7 macrophages upon incubation with LPS and the compound mixtures. In addition, no membrane disruption was observed in bacterial cells treated with the diastereomeric mixtures, whereas polymyxin B showed significant membrane disruption.

To more specifically study the stereochemical requirements of the polymyxin mechanism, we recently reported the first total synthesis of the unnatural enantiomer of full-length polymyxin B_4_, *ent*-PmxB_4_,^96^ as well as the previously reported *ent*-PMBN.^98^ When tested against a panel of Gram-negative bacteria we observed essentially no activity for *ent*-PmxB_4_ in MIC assays (MICs ≥ 128 μg/mL). This is in notable contrast to the activity reported for the stereorandomized mixtures of polymyxin B diastereomers described by Reymond and co-workers.^99^ The above-mentioned findings clearly indicate that while some (combinations of) residues are amenable to stereochemical inversion, doing so for all amino acids is not tolerated. Also of note was our finding that despite lacking inherent activity, *ent*-PmxB_4_ does effectively synergize with rifampicin against *E. coli* ATCC 25922 (FICI: 0.08). When assessing OM permeabilization by means of NPN assays, we also found that PmxB_4_ and PMBN, with natural stereochemistry, were more effective than their corresponding enantiomers. Interesting, however, was the finding that for *mcr*-1 positive *E. coli*, the NPN uptake is rather similar for PMBN and *ent*-PmxB_4_, indicating that for polymyxin resistant strains, the addition of a lipophilic tail to the unnatural enantiomer can compensate for the otherwise reduced interactions with LPS. In the final analysis, it is evident from our studies and those of others, that stereochemical considerations play a key role in the mechanisms and activities of the polymyxins.

## Development of Polymyxin Analogues with Reduced Nephrotoxicity

Given the dose-limiting toxicity associated with the clinical use of polymyxins, much effort has been spent in trying to develop analogues with improved therapeutic indices. In recent years a small number of new polymyxins have entered clinical trials while other analogues also offer insights in the nephrotoxicity of this class of compounds. Here, we summarize these developments in three distinct subsets including: (1) new polymyxins currently undergoing clinical development; (2) recent polymyxin analogues reported with accompanying antibacterial activity and nephrotoxicity data but not (yet) in clinical development; and (3) novel analogues for which structures have recently been disclosed but without extensive antibacterial activity or nephrotoxicity data. For reasons of convenience, we refer to the compounds according to their original codes or numbers used in the source literature cited. To provide the reader with a concise overview of the various methods in the synthesis of the different polymyxins here covered, we include summary Table 1 at the end of the review. Also, to allow for convenient comparison, we provide a tabular summary of the antibacterial activities and associated toxicities for all polymyxin analogues here covered (Table 2).

**Table 1:**
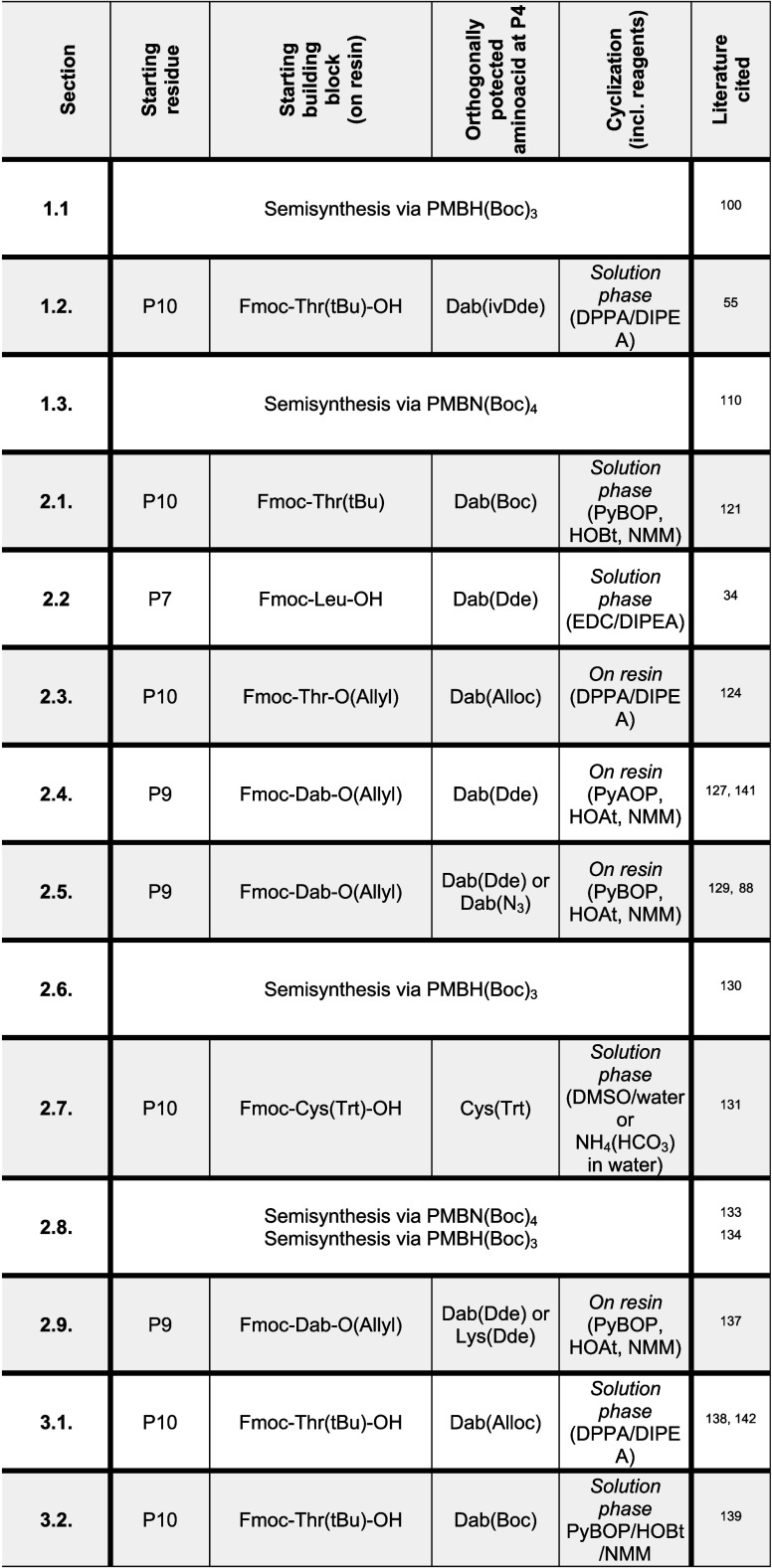
Summary of Recent (2010−) Strategies in Polymyxin Synthesis, Encompassing Both Semi- and Total Synthesis Approaches

**Table 2:**
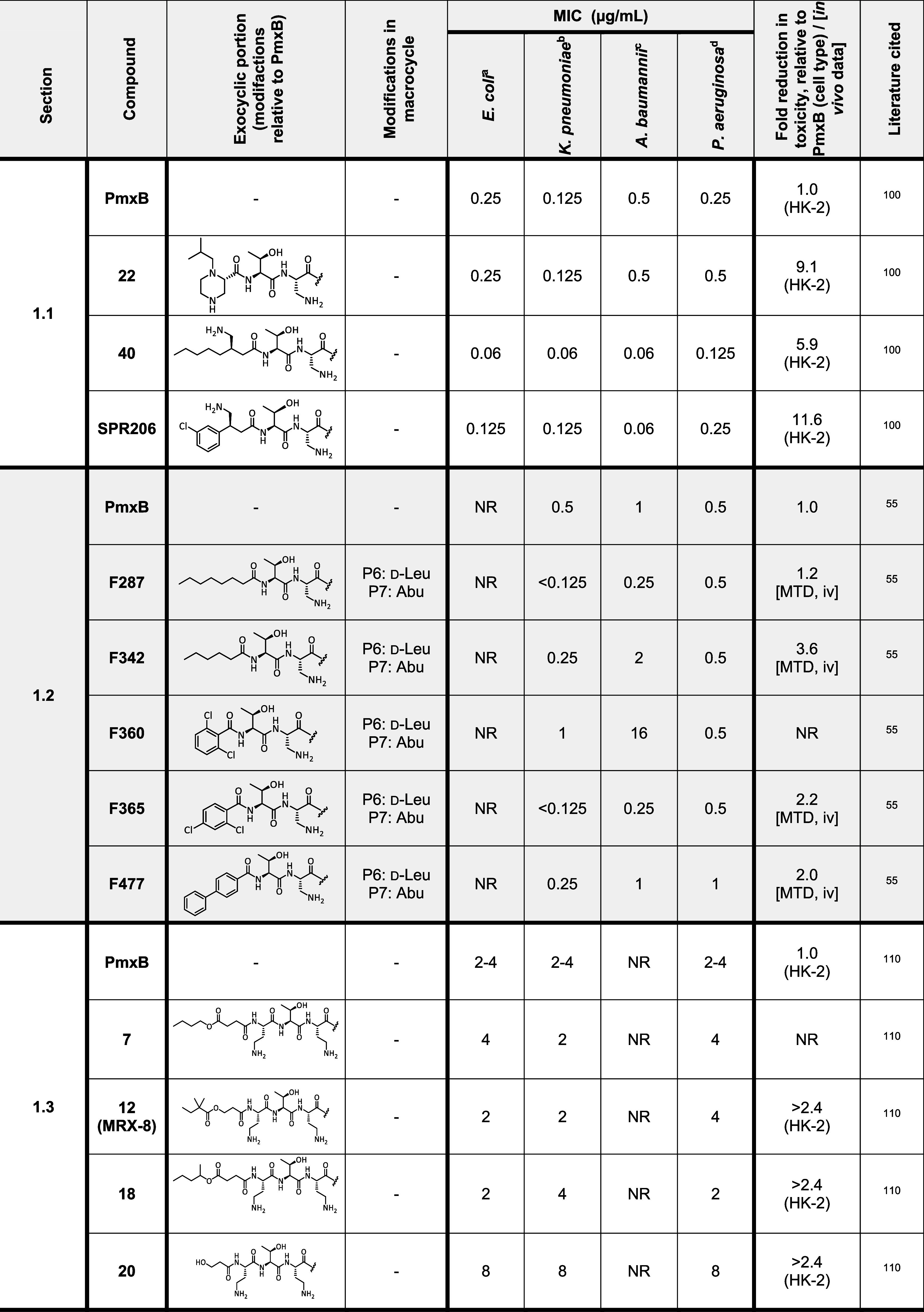

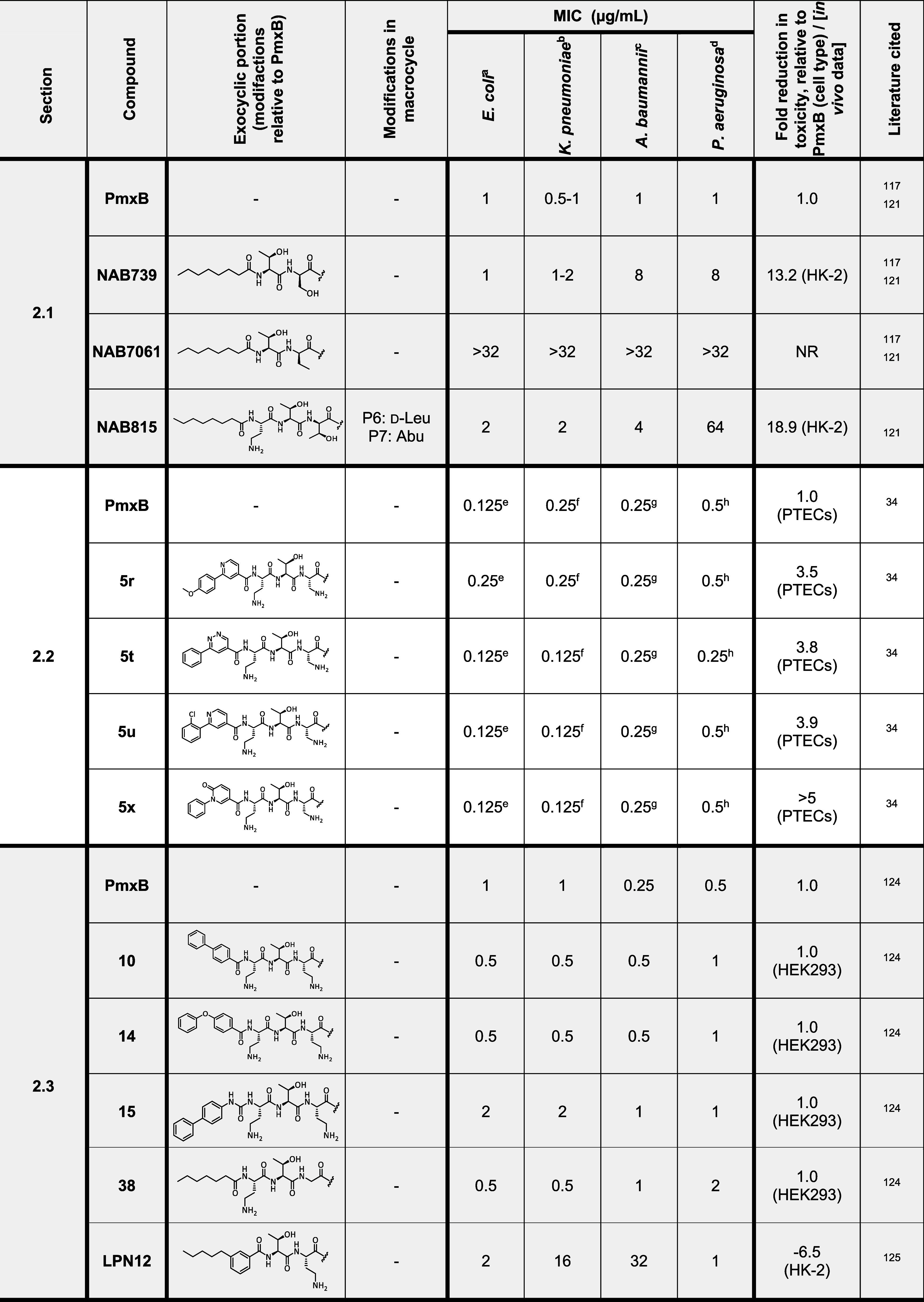

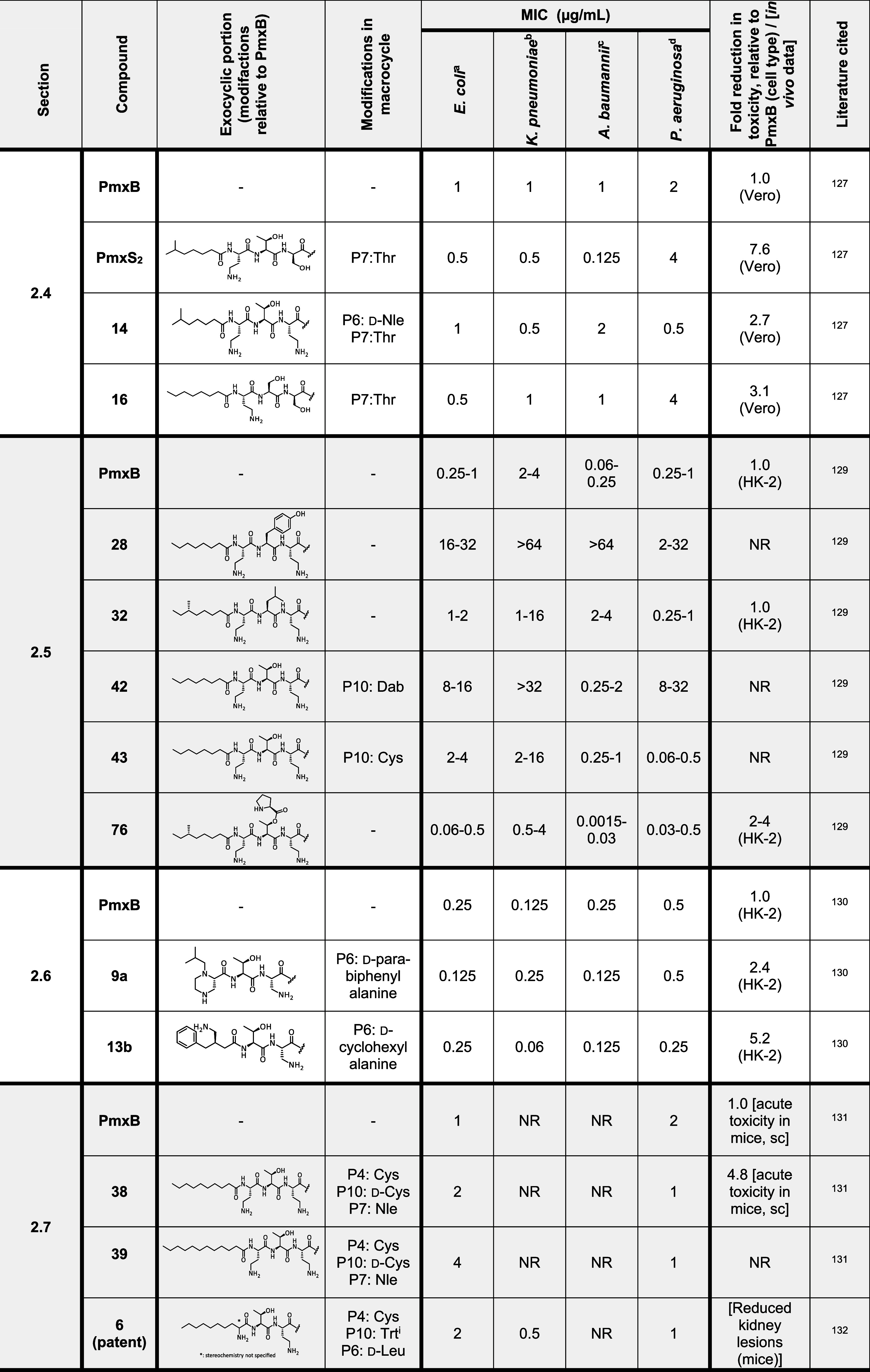

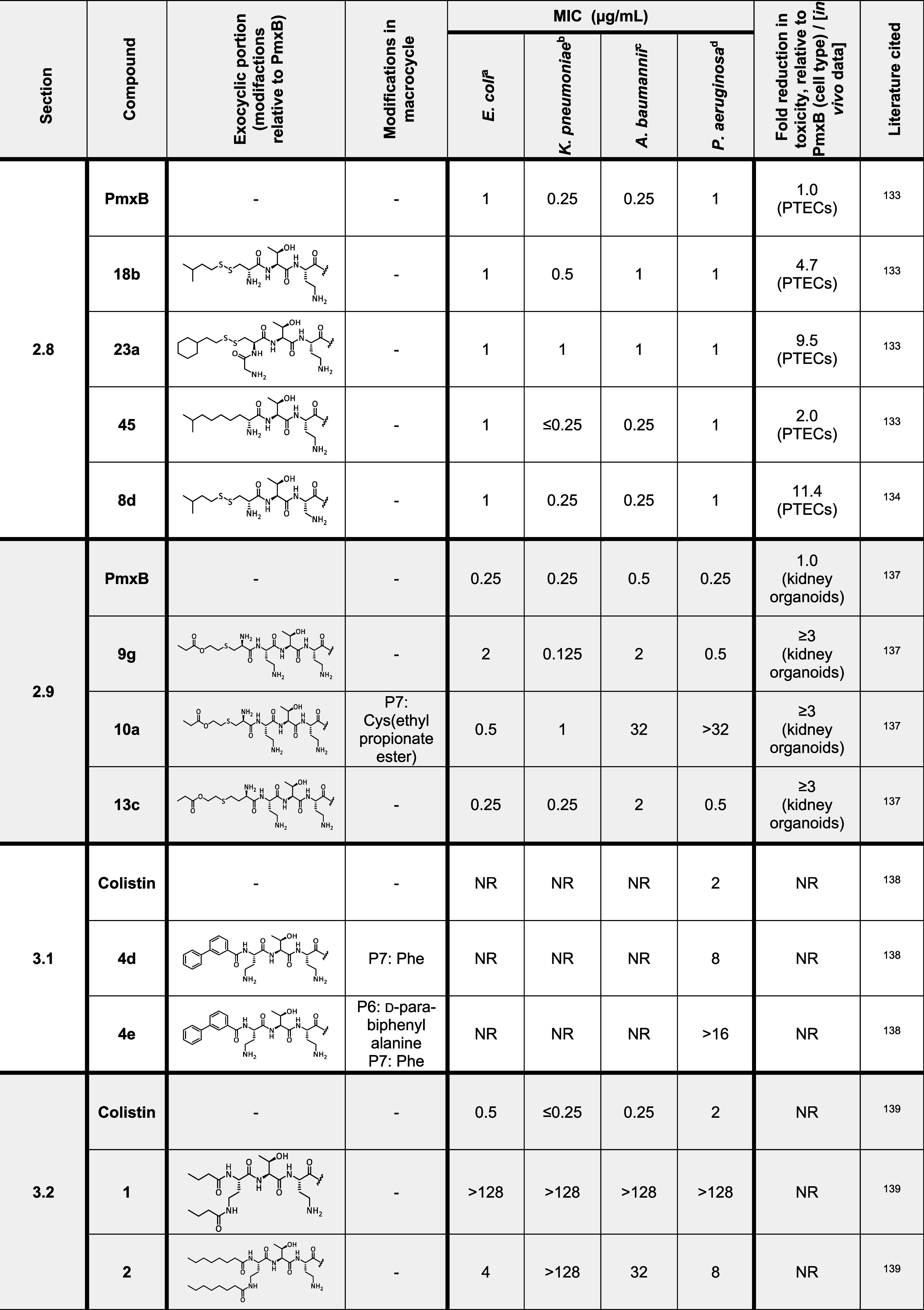
Comparative Overview of Selected Polymyxin Analogues, Including Representative MIC Values and Nephrotoxicity Data

a ATCC 25922.

b Various strains.

c ATCC 17978 or ATCC 19606.

d ATCC 27853.

e Strain EC-1.

f Strain KP-3700.

g Strain AB-3167.

h Strain PA-01.

i Trt: (2*R*,3*S*)-3-amino-4-mercaptobutan-2-ol, connected via amine and thiol (disulfide forming). Abbreviations: NR = Not reported; MTD = maximal tolerated dose; iv = intravenous; sc = subcutaneous; PmxB = polymyxin B. Noncanonical amino acids: Abu = amino butyric acid; Dab = diamino butyric acid; Nle = norleucine.

## 1 Polymyxin Analogues in Clinical Development

### 1.1 SPR206

**Scheme 1:**
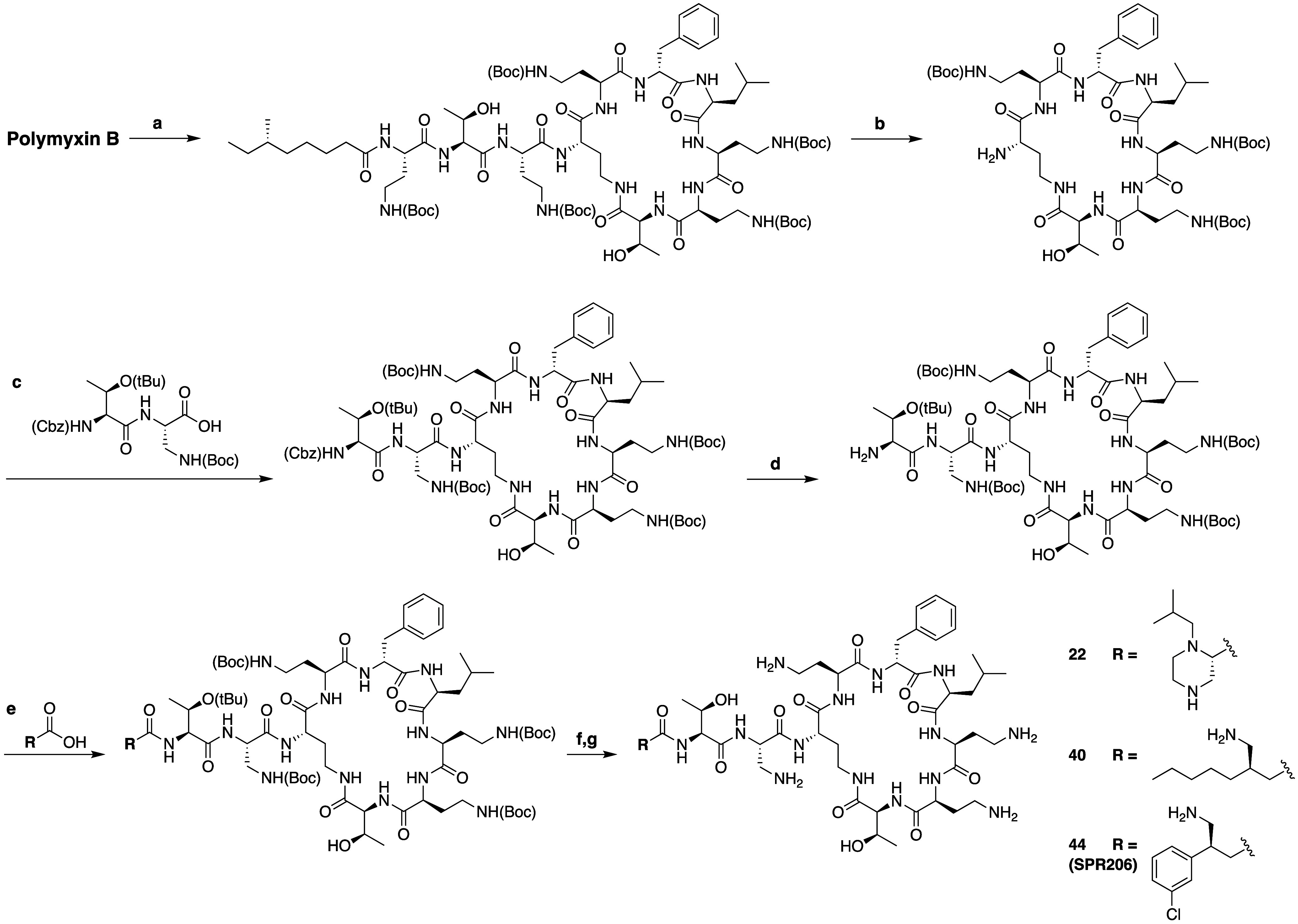
Semisynthetic Route Used by Spero Therapeutics to Access Novel Polymyxin B Analogues Reagents and conditions: (a) (Boc)_2_O, Et_3_N, CH_3_CN/H_2_O; (b) Savinase, CH_3_CN/H_2_O; (c) HATU, DIPEA, DCM/DMF; (d) 10% Pd/C, MeOH; (e) HATU, DIPEA, DCM/DMF; (f) TFA/DCM; (g) Purification by RP-HPLC.

Spero Therapeutics is currently pursuing clinical development of its lead polymyxin candidate **SPR206** which was selected from a series of novel polymyxin analogues bearing structure variations in the exocyclic moiety, specifically modifications of the P1 and P3 residues and *N*-terminal acyl group (Scheme 1).^100^ These analogues were prepared via an established semisynthetic route wherein all five amine groups of polymyxin B were first Boc protected followed by enzymatic degradation using Savinase to yield the corresponding tri-Boc protected polymyxin B heptapeptide PMBH(Boc)_3_.^101^ Subsequent coupling with the dipeptide building block Cbz-Thr(tBu)-Dap(Boc), followed by selective removal of the Cbz group, provided an intermediate that was then further diversified by addition of different acyl groups followed by Boc group removal. Notable among these polymyxin analogues is the omission of the P1 residue. Rather, the Spero team found that the amine normally present in the side chain of the Dab residue at P1 could instead be incorporated into the acyl group appended to the *N*-terminus leading to the identification of compound **22** as a particularly potent analogue that also exhibited reduced *in vitro* kidney cell toxicity against HK-2 cells. However, the reduced cell toxicity of **22** did not translate to a reduced nephrotoxicity when assessed *in vivo* in mice. By analyzing the accumulation of the compound in the kidney, it was found that the kidney exposure of compound **22** in fact far exceeded that of polymyxin B.

In an attempt to address this liability, the Spero team next explored the effect of shifting the position of the free-amine present in the *N*-terminal acyl group. This led to analogues **40** and **44**, which displayed an improved balance of potency, toxicity and kidney exposure. In particular, compound **44** (now known as **SPR206**) was found to possess antibacterial activity and kidney exposure on par with that of polymyxin B, while exhibiting a 12-fold lower cytotoxicity toward HK-2 cells.

When tested against a panel of bacteria (200 strains), **SPR206** was found to be broadly active, consistently displaying MIC_50_ values below 1 μg/mL against all Gram-negative members of the ESKAPE family. In general, **SPR206** outperformed polymyxin B and colistin, with MIC values 2- to 4-fold lower. Against a subset of *A. baumannii* strains (33 strains, including drug resistant isolates), an impressive MIC_90_ of 0.125 μg/mL was reported.^102^ In a neutropenic murine thigh infection model, **SPR206** exhibited efficacy on par with that of polymyxin B, resulting in a 7-log_10_ (CFU/mL) reduction in viable bacteria when dosed at 0.86 mg/kg. Furthermore, in a neutropenic murine pneumonia model, **SPR206** again outperformed polymyxin B, showing a 2-log_10_ (CFU/mL) reduction of viable *A. baumannii* NCTC 13301 at a concentration where polymyxin B showed no effect (17.2 mg/kg). Spero recently published the results of a Phase I clinical trial with **SPR206**.^103^ When tested in a population of healthy volunteers, no evidence of nephrotoxicity was observed upon dosing at 100 mg q8h for 14 days. These encouraging results lay the foundation for a Phase II trial, currently in the planning stages, wherein the capacity for **SPR206** to treat multidrug resistant (MDR) Gram-negative bacterial infections, specifically hospital-acquired and ventilator-associated bacterial pneumonia (HABP/VABP), will be evaluated.^104^

### 1.2 QPX9003

**Scheme 2:**
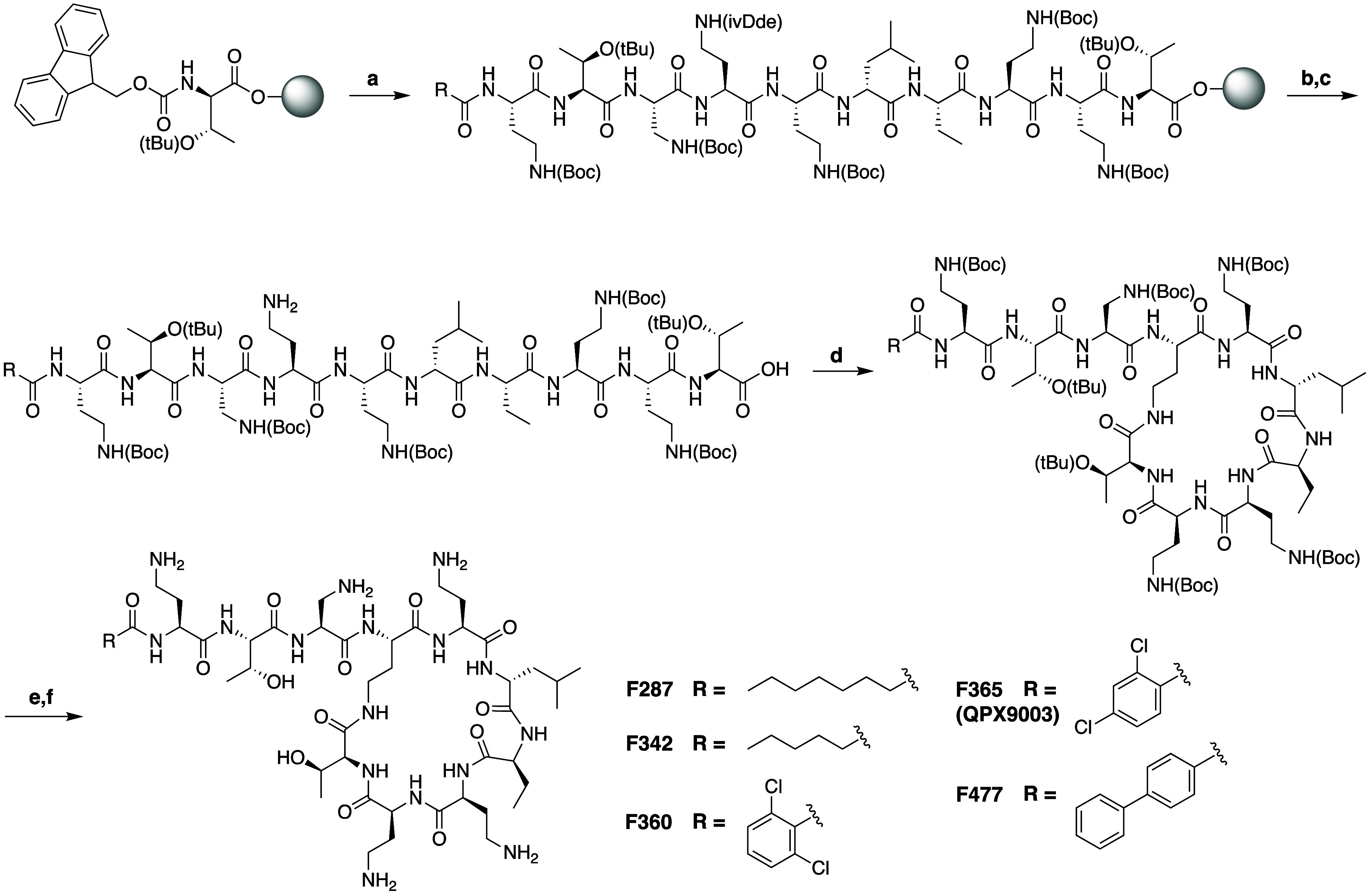
Synthesis of **QPX9003** and Related Analogues Reagents and conditions: (a) 10 SPPS cycles: (i) deprotection (20% piperidine/DMF); (ii) coupling (Fmoc-AA, HCTU, DIPEA (1:1:2); (b) 3% N_2_H_4_/DMF; (c) HFIP/DCM; (d) DPPA, DIPEA; (e) TFA/TIPS/EDT; (f) Purification by RP-HPLC.

The groups of Li and Velkov recently reported the development of a novel synthetic polymyxin analogue with promising safety and efficacy against lung infections caused by Gram-negative pathogens.^55^ Originally assigned the compound code **F365**, this polymyxin analogue was later renamed as **QPX9003** by QPEX Biopharma (recently acquired by Shionogi) who licensed it for clinical development. **QPX9003** resulted from a focused SAR study which investigated the effect of modifications of the *N*-terminal hydrophobic tail, the P3 residue, and the hydrophobic patch associated with the P6/P7 residues. Substitution of residues present in the polymyxin macrocycle preclude semisynthetic approaches and as such **QPX9003** and the other analogues prepared were fully assembled by solid phase peptide synthesis (SPPS), starting with Fmoc-Thr(tBu)-OH loaded onto CTC resin (Scheme 2).^105^ The side chain of Dab at P4 was protected with an orthogonal ivDde protecting group, which is conveniently removed on resin by treatment with hydrazine solution after assembly of the complete linear polymyxin precursor. Subsequent cleavage from the resin using mild acidic condition to maintain all other side chain protection was followed by solution phase cyclization with DPPA/DIPEA. The final step involved global deprotection and HPLC purification to yield the desired polymyxin species.

Antibacterial assays with the polymyxin analogues generated using this approach revealed that amino acids at P6 and P7 could be exchanged for less hydrophobic residues (e.g., by changing Phe or Leu for Val or Abu), with analogues containing the d-Leu-Abu motif at P6/P7 maintaining the activity of polymyxin B. Based on previous reports describing the benefit of modification at P3,^34,100^ the Dab residue at this position was replaced by Dap, to give analogues with increased potency and decreased toxicity. Finally, the *N*-terminal acyl moiety was optimized starting with saturated linear lipids as in analogue **F287** bearing an octanoic acid tail found typically in polymyxins. **F287** was found to exhibit potent activity, with lower MICs than polymyxin and it was proposed to be less nephrotoxic as a consequence of reduced kidney cell membrane penetration, as predicted by MD approaches.^106^ However, the maximum tolerated dose (MTD) for **F287** was found to be rather low and similar to polymyxin. Analogue **F342**, with a shorter C6 lipid, was shown to be better tolerated (by ca. 2.5-fold), but exhibited reduced activity against *A. baumannii* strains. This led to evaluation of a new series of analogues including **F360**, **F365**, and **F477**, all bearing alternative aromatic acyl groups, given that similar *N*-terminal moieties had previously shown benefit in other polymyxin optimization programs.^34,100,107^ Among the analogues thus prepared, the 2,4-dichloro benzoyl substituted variant (**F365/QPX9003**) was found to possess the best balance of activity with low acute toxicity. Interestingly, **QPX9003** exhibited consistently lower MIC values compared to the highly similar 2,6-dichloro benzoyl analogue **F360**. Overall, the ratio between MIC and MTD proved to be optimal for **QPX9003** and in a blood infection model in neutropenic mice the compound was found to outperform colistin in reducing bacterial burden 4 h post treatment.^55^

To estimate the efficacy of **QPX9003** in a lung infection model, MICs were determined in the presence of Survanta, a natural bovine lung surfactant. Sputum derived biomolecules, including surfactants, have previously been shown to negatively impact the activity of lipopeptide antibiotics.^108^ Notably, while the MIC values measured for polymyxin B were found to be elevated by as much as 10-fold in the presence of 10% Survanta, no such increase was observed for **QPX9003**. This effect can be attributed to the reduced hydrophobicity of the Abu residue present at P7 in **QPX9003** as opposed to the Leu residue found at the same position in polymyxin B.^55^*In vivo* studies showed **QPX9003** to have lower plasma protein binding and increased urinary recovery relative to polymyxin B. These findings suggest that the compound is taken up by proximal tubule epithelial cell (PTECs) to lesser extent, which might also contribute to its reduced toxicity. Furthermore, MS imaging revealed a relatively limited kidney distribution for **QPX9003**, primarily restricted to the renal cortex. In addition, genetic analysis on HK-2 cells showed that **QPX9003** only impacted the expression patterns for 70 genes, compared to a much larger number of 1282 genes upon treatment with colistin. As shown by Li’s earlier work,^54^ HK-2 cells with a *KNCJ16* (inwardly rectifying potassium channel Kir5.1) knockout are generally protected from the toxic effects of lipopeptides. For **QPX9003**, no differences are seen for the parent cell line and the knockout, suggesting that the compound does not affect the inwardly rectifying potassium channel Kir5.1.^55^

The development of **QPX9003** represents a promising new polymyxin design strategy to access analogues with enhanced properties. Notably, reduction in overall charge does not appear to be an obligate requirement for achieving reduced nephrotoxicity. A Phase I trial run in 2022 with **QPX9003** substantiated the safety of the compound, paving the way for its continued clinical development.^109^

### 1.3 MRX-8

**Scheme 3:**
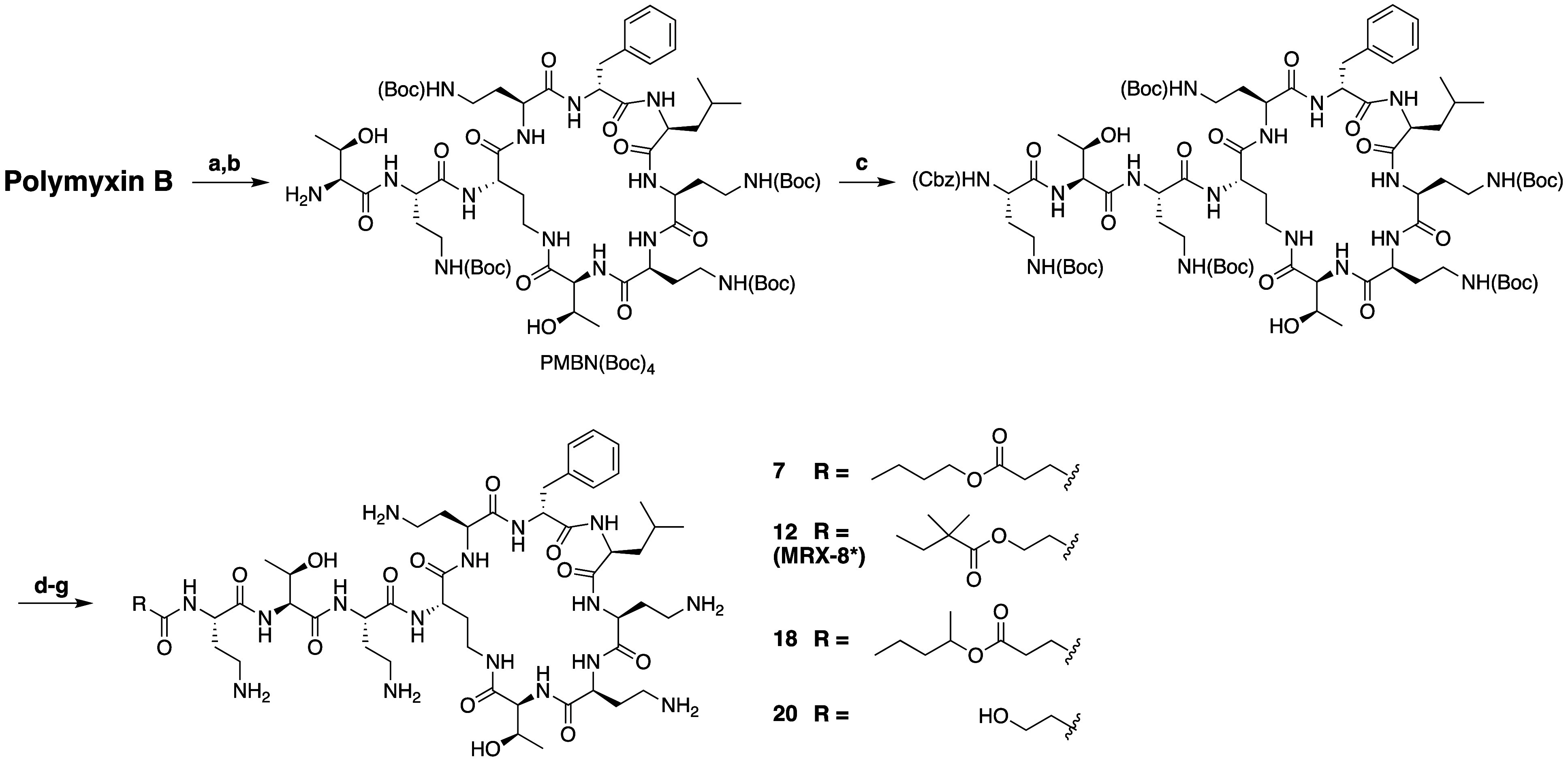
Synthesis of N-Terminal Lipid Ester-Linked Polymyxin Analogues Developed by MicuRx Pharmaceuticals Reagents and conditions: (a) Ficin, DTT, H_2_O; (b) (Boc)_2_O, Et_3_N; (c) Cbz-Dab(Boc)-OH, HATU, DIPEA, DCM/CH_3_CN; (d) 10% Pd/C, MeOH; (e) RCO_2_H, HATU, DIPEA, DCM/CH_3_CN; (f) TFA/H_2_O/TES; (g) Purification by RP-HPLC. *Presumed structure.

Recently, Shanghai-based MicuRx Pharmaceuticals disclosed a series of novel polymyxin analogues containing metabolically labile ester moieties in their *N*-terminal tail moiety (Scheme 3).^110^ The lead compound being taken forward for clinical development is designated as **MRX-8** and while its specific structure has not been confirmed by MicuRx, based on the breadth of patent data specifically provided for analogue **12** (see Scheme 3) it seems likely that this is the structure of the compound. As noted, **MRX-8** and related analogues were designed to be metabolically labile. Specifically, by incorporation of an ester linkage in the lipid tail, these polymyxins are designed to be degraded *in vivo* by blood plasma esterases, resulting in the formation of metabolites with reduced nephrotoxicity. The synthesis of these analogues is achieved by means of an established semisynthetic route wherein polymyxin B is first selectively degraded using the proteolytic enzyme ficin to remove the *N*-terminal Dab residue and lipid moiety, followed by full Boc protection to yield the PMBN(Boc)_4_ building block (Scheme 3).^111^ Next, the Dab residue is reinstalled at P1 by coupling of the orthogonally protected Cbz-Dab(Boc)-OH, followed by hydrogenation to yield the corresponding intermediate with a free *N*-terminus. This protected decapeptide in turn allows for convenient diversification through coupling of various acyl groups featuring an internal ester motif.^110^

The analogues depicted in Scheme 3 were found to exhibit potent antibacterial activity on par with that of polymyxin B. It was subsequently shown that incubation of the compounds with plasma resulted in significant degradation of analogues **7** and **18** with only 15% and 18% remaining respectively after 4 h incubation while no degradation of polymyxin B was detected. By comparison to **7** and **18**, analogue **12**/**MRX-8** showed greater plasma stability with 31% of the compound intact after 4 h incubation. These findings indicate that both the regiochemistry of the ester linkage as well as the flanking substituents play a key role in the stability of these compounds. The (enzymatically) hydrolyzed product (Scheme 3, denoted as compound **20** in the reporting patent) bears a hydroxyethyl motif and retains moderate activity against *P. aeruginosa*, *E. coli*, and *K. pneumoniae* (2–4-fold increase compared to the parent compound **MRX-8**, Table 2).^110^

When tested on a large set (*n* = 1314) of clinical isolates, **MRX-8** was found to have consistently high potency with MIC_90_ values of 0.25, 0.25, 1.0, and 1.0 μg/mL against *E. coli*, *K. pneumoniae*, *A. baumannii*, and *P. aeruginosa*, respectively (versus 0.5, 0.5, 0.5, 1 μg/mL for polymyxin B against the same strain collection).^112^ Similar efficacy was also observed in a separate study recently reported.^113^ Based on these promising *in vitro* data, **MRX-8** was taken forward for preclinical development with similarly encouraging results.^114^ At present, MicuRx Pharmaceuticals is conducting Phase I clinical trials to assess the safety, tolerability, and pharmacokinetic profile of **MRX-8** when administered intravenously in healthy subjects.^115^

While more than a decade has passed since the last human trials were run with a novel polymyxin, at present three new candidates are under clinical investigation. Prior to these, the last polymyxin to be evaluated in Phase I trials was Cubist Pharmaceutical’s polymyxin B analogue **CB-182 804**, whose development was stopped in 2010 due to toxicity concerns.^116^ In this light, the clinical candidates currently under investigation have all been carefully scrutinized, particularly for nephrotoxicity. In the case of **SPR206**, the limited renal uptake is likely to be a major advantage, while **QPX9003** appears to have a relatively high tolerated dose with a favorable kidney safety profile. For **MRX-8**, the short decomposition half-life, and the presumed lower toxicity of the resulting metabolites, provides the foundation for its enhanced therapeutic profile. The progress of these three clinical candidates will be closely watched in the coming years in the hope that they will offer physicians and their patients with safer and effective options for polymyxin therapy.

## 2 Polymyxin Analogues with SAR and (Nephro)Toxicity Data

### 2.1 Analogues with Reduced Charge

**Figure 2:**
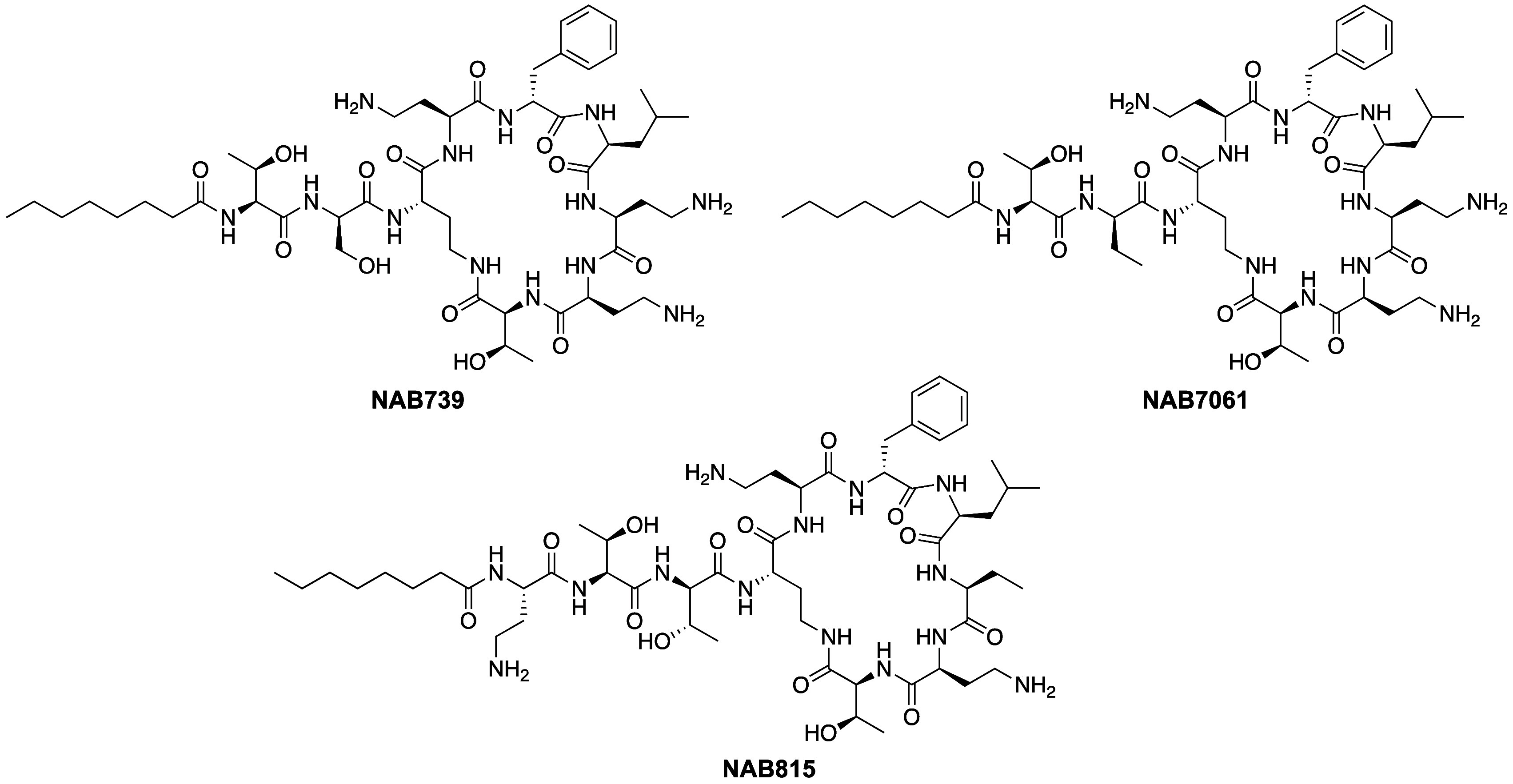
Polymyxin analogues developed by Northern Antibiotics featuring a reduced net positive charge at physiological pH.

Based on the hypothesis that the polycationic nature of the polymyxins contributes significantly to their toxicity, researchers at the Finnish company Northern Antibiotics explored a series of polymyxin analogues containing a reduced number of cationic residues.^117^ These analogues were prepared by solid-phase synthesis. Common to some of the more potent analogues identified by the Northern Antibiotics team was the presence of d-Ser rather than Dab at P3, a substitution also found in a subset of the natural polymyxins including polymyxin D,^118^ S,^119^ and macolacin^25^ (see Figure 1A above). Further reduction of the overall cationic charge by removal of the Dab residue at P1, in combination with the d-Ser for Dab substitution at P3, gave polymyxin B analogue **NAB739** (Figure 2) that was found to retain significant antibacterial activity. Against *Enterobacteriaceae*, **NAB739** exhibits activity similar to polymyxin B, while against strains of *Acinetobacter* and *Pseudomonas* polymyxin B was found to be superior.^117^ Also of note was the finding that the hydroxy group of d-Ser at P3 is essential for potency, with a d-Ser to d-Abu substitution as in **NAB7061** (Figure 2) resulting in a large drop in activity.^120^ The impact of the reduced net positive charge of these polymyxins on their nephrotoxicity was also assessed. These studies showed that they exhibit reduced binding to the brush border membrane of the rat renal cortex and also display significantly reduced toxicity against HK-2 cells (Table 2).^117,121^

Whereas **NAB739** bears all 3 cationic charges in its macrocycle and none in the exocyclic moiety, novel analogue **NAB815** (prepared by total synthesis), containing d-Thr at P3, was designed to contain one positive charge in the exocyclic region and only two in the macrocycle (Figure 2).^121,122^**NAB815** was found to have the same antibacterial activity as **NAB739** (MIC of 2 μg/mL against *E. coli* ATCC 25922, Table 2) with both being slightly less active than polymyxin B (0.5 μg/mL on *E. coli* ATCC 25922). Surprisingly, the CC_50_ value determined for **NAB815** with HK-2 cells (334 μg/mL) was found to be about 20-fold higher than that of polymyxin B (18 μg/mL) and 1.4 times higher compared to **NAB739** (237 μg/mL).

Also of note is the finding that the NAB analogues are effectively secreted via the urine, a property not observed for most natural polymyxins.^122^ This feature was hypothesized to be a consequence of their lower uptake by PTECs resulting in a reduced accumulation in the kidneys. The accompanying increase in renal secretion might also qualify these compounds for treatment of urinary tract infections. Despite their higher *in vitro* MICs relative to polymyxin B, both **NAB739** and **NAB815** demonstrated superior performance in a pyelonephritis infection model with *E. coli*, requiring only about 10% of the amount of polymyxin B to achieve the same effect.^123^

### 2.2 Analogues Containing P3 Substitutions and *N*-Terminal Biaryl Moieties

**Figure 3:**
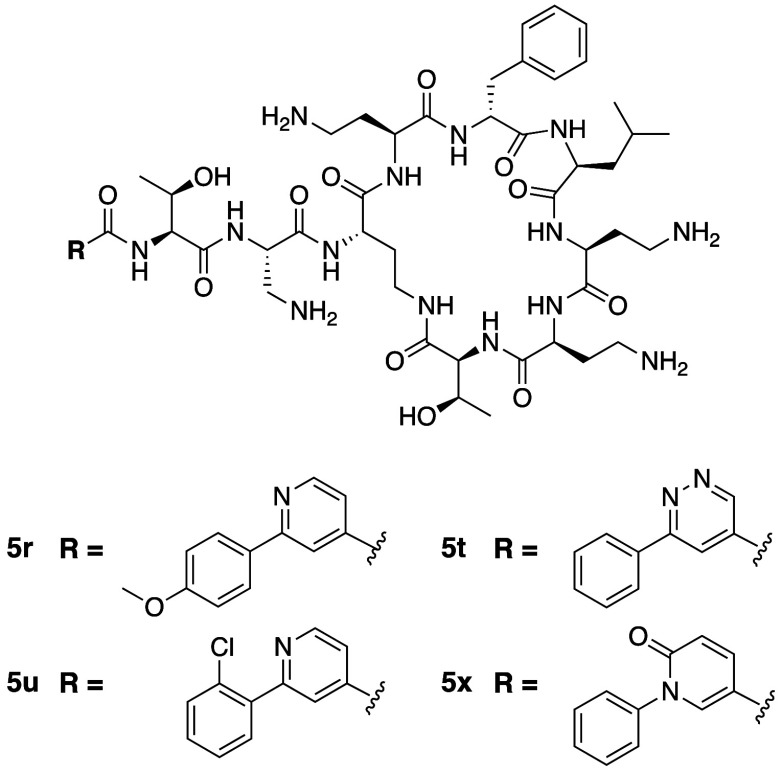
Polymyxin B analogues developed by Pfizer containing P3 Dap for Dab substitution and various biaryl moieties at the N-terminus.

Researchers at Pfizer employed a total synthesis approach to generate a series of novel polymyxin B analogues with various amino acids at P3 along with the introduction of different biaryl moieties at the *N*-terminus conjugated via amide, urea, or sulfonamide linkages.^34^ These studies led to the important finding that replacement of the P3 Dab residue normally found in polymyxin B with the shorter Dap resulted in both increased antimicrobial activity as well as a >2-fold reduced toxicity toward PTECs. Subsequent screening of various biaryl moieties at the *N*-terminus resulted in a panel of novel analogues with potency equivalent to polymyxin B. While many of these analogues also exhibited CC_50_ values similar to polymyxin B against PTECs, a subset was found to display >2-fold reduced toxicity, including compounds **5r**, **5t**, **5u**, and **5x** (Figure 3, Table 2). Among these, compound **5x** was identified as the clear favorite, displaying a CC_50_ value >5-fold higher than that of polymyxin B. Compound **5x** was also found to be equipotent to polymyxin B, with IC_90_ values of 2 μg/mL against *E. coli*, *P. aeruginosa*, and *A. baumannii*, while against *K. pneumoniae* its IC_90_ of 1 μg/mL was lower than that of polymyxin B (2 μg/mL). Also noteworthy is the activity of **5x** against drug-resistant *A. baumannii* isolates where its IC_90_ value was found to be 16 μg/mL, compared to >64 μg/mL for polymyxin B. Disappointingly, while **5x** showed reduced toxicity toward PTECs and in exploratory rat studies, results from dog studies did not demonstrate superiority to polymyxin, leading to the termination of its development.

### 2.3 Analogues Assessing Substitution of All Positions and *N*-Terminal Acyl Group Variation

The group of Blaskovich and Cooper reported a comprehensive polymyxin structure–activity study assessing the contribution of each variable amino acid position (excluding Dab at P4 and Thr at P10 as they are involved in ring closure) and variations of the *N*-terminal acyl moiety.^124^ Using a total synthesis approach, a large number of new polymyxin analogues were prepared. While many of the amino acid substitutions sampled resulted in reduced potency, some modifications to the lipophilic tail, such as the inclusion of aromatic biphenyl moieties, led to analogues that retained potent activity. In line with the findings of the Pfizer team (see previous section) the introduction of d-Ser and d-Dab at P3 were also found to be well tolerated. In addition, a novel analogue bearing Gly at P3 was found to exhibit the same activity as polymyxin B against *Enterobacteriaceae* strains. By comparison, P5 and P9 were found to be least amendable to modification, resulting in significant loss of activity upon the introduction of relatively minor structural changes. Notable was the effect of increased hydrophobicity of the side chains at P6 and P7 which resulted in a slight enhancement of activity against polymyxin resistant strains. These analogues, however, also exhibited decreased CC_50_ values on HepG2 cells, suggesting nonbacteriospecific cytotoxicity. The residue at P8 was also found to be somewhat tolerant of modification with an Arg for Dab substitution at this position resulting in an analogue with activity comparable to polymyxin B.

**Figure 4:**
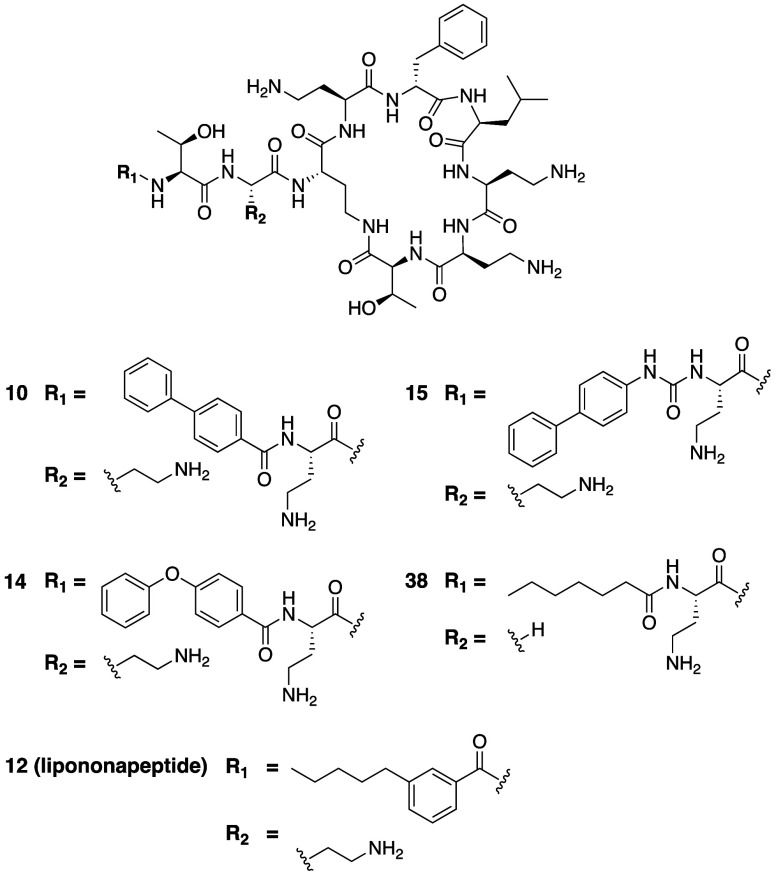
Polymyxin B analogues developed by Blaskovich and Cooper to investigate substitution at P3 and variation of the N-terminal acyl group.

Based on the results of antibacterial assays, analogues **10**, **14**, **15**, and **38** (Figure 4, Table 2) were selected for further cytotoxicity testing on HEK293 cells which indicated that they are not more toxic than polymyxin B. In addition, Lactate Dehydrogenase (LDH) and γ-Glutaryl Transferase (GGT) release assays on primary cells revealed significantly increased CC_50_ values for compounds **10**, **14**, **15** and **38** compared to polymyxin B. Specifically, the compounds were all found to exhibit higher CC_50_ values for LDH release (CC_50_ ≥ 122 μg/mL) compared to polymyxin B (CC_50_ 23 μg/mL) while for the GGT release assay the reported CC_50_ values for the analogues was >128 μg/mL compared to 177 μg/mL for polymyxin B.

The same group also evaluated a series of lipononapeptide polymyxin B analogues lacking the Dab at P1, prepared via a total synthesis strategy similar to that used for their previous full-length analogues.^124,125^ In doing so a number of lipidated PMBN variants were generated by incorporating various *N*-terminal acyl moieties. Omitting the Dab at P1 along with *N*-terminal acylation with octanoic acid led to a 2–4-fold increase in MIC values against a panel of Gram-negative pathogens tested. The incorporation of structurally diverse acyl groups gave rise to novel analogues such as compound **12** (Figure 4) that against some strains showed antibacterial activity approaching that of the parent polymyxin. However, the most active lipidated PMBN analogues thus identified were also found to exhibit significantly higher cytotoxicity toward HK-2 cells compared to polymyxin B.

### 2.4 Analogues Based on Previously Uncharacterized Natural Polymyxins

**Figure 5:**
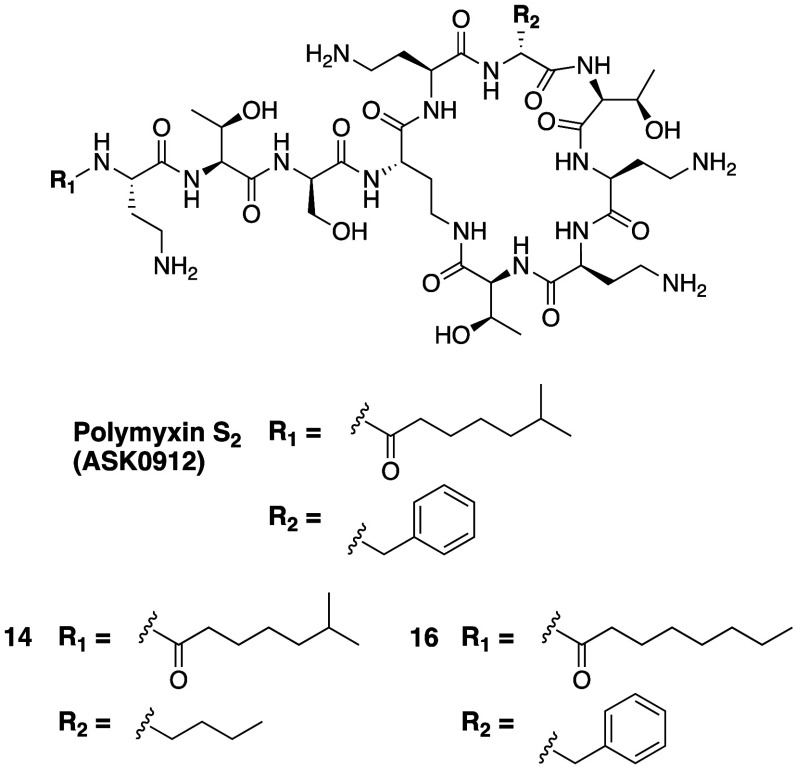
Polymyxin S_2_ analogues (containing d-Ser at P3 and Thr at P7) developed by the group of You and Li.

The group of You and Li undertook an extensive comparative analysis of the activity and toxicity of a panel of natural polymyxin variants including a number of previously unexplored derivatives.^126,127^ Of the 29 natural polymyxins studied, polymyxin T variants, containing Leu at P10, showed the weakest antibacterial activity, together with high toxicity toward Vero cells. All other natural polymyxins were found to exhibit potent activity (MICs on *E. coli*: ≤ 2 μg/mL; *K. pneumoniae*: ≤ 2 μg/mL; *A. baumannii*: ≤ 2 μg/mL and *P. aeruginosa:* ≤ 4 μg/mL). Interestingly, the natural polymyxin B variants included in the study showed the highest toxicity toward Vero cells while the previously unreported polymyxin A_2_, D_2_, and S_2_ showed the lowest toxicity. The latter three analogues contain the same branched 6-methyl heptanoyl tail, indicating that the slightly shorter lipid offers a benefit in reducing toxicity compared to polymyxin variants that bear the 6-methyl octanoyl moiety. These studies highlight the value of exploring scaffolds other than polymyxin B, as has traditionally been the case in medicinal chemistry approaches aimed at finding improved analogues. This assertion is supported by the discovery that polymyxin S_2_, containing d-Ser at P3 and Thr at P7 (Figure 5, Table 2), was found to exhibit potent antibacterial activity comparable to that of polymyxin B but with a 7-fold lower cytotoxicity. Unnatural analogues of Polymyxin S_2_ were subsequently prepared to explore the impact of changes to the P6 position as for compound **14**, where d-norleucine was incorporated at P6, as well as the effect of linearization of the *N*-terminal lipid as in analogue **16** (Figure 5). While **14** and **16** both exhibit potent antibacterial activity, they were not found to be less toxic than the parent polymyxin S_2_. Based on these findings, polymyxin S_2_ was further evaluated in a murine model of systemic infection with NDM-1 positive *K. pneumoniae*. This revealed an effective dose (ED_50_) of 0.9 mg/kg for polymyxin S_2_ compared with 2.2 mg/kg for polymyxin B, in agreement with their respective *in vitro* MIC values.^127^ Currently under development as ASK0912, polymyxin S_2_ was also recently evaluated in a murine model of *A. baumannii* septicemia, where it again showed an overall better therapeutic potential (lower required dose, better bacterial clearance) compared to polymyxin B.^128^

### 2.5 Analogues Designed to Explore the Roles of Conserved Thr at P2 and P10

**Figure 6:**
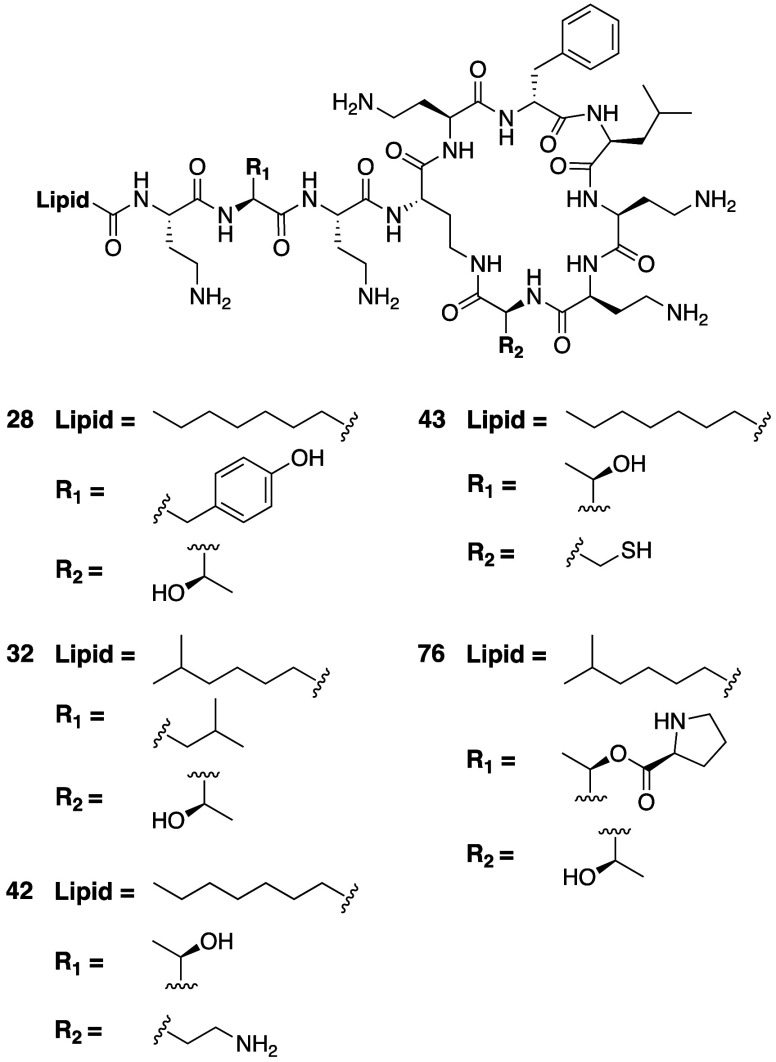
Polymyxin analogues prepared by Huang and co-workers to probe the roles of Thr residues at P2 and P10.

Huang and co-workers reported a study focused specifically on characterizing the contributions of the Thr residues found at P2 in all known natural polymyxins and at P10 in the vast majority of polymyxins.^129^ In doing so they relied on both semisynthesis and total synthesis approaches. These investigations revealed that the Thr at P2 is only modestly tolerant to substitution, with most P2 substituted analogues showing increased MIC values against most of the bacterial strains tested. The one exception was in the case of *P. aeruginosa*, where a select few P2 substitutions were found to yield polymyxin analogues that showed the same activity as polymyxin B. This effect is rather amino acid specific with analogue **32**, bearing Leu at P2 (Figure 6, Table 2), maintaining potent anti-*P. aeruginosa* activity along with slightly elevated MICs against the other species tested, while analogue **28** with Tyr at P2 exhibited a significantly reduced activity against all strains. In contrast, the Thr residue at P10 was found to be amenable to substitution by a broader range of amino acids (Cys, Val, Leu, Phe), while keeping good activity against both *P. aeruginosa* and *A. baumannii* (MICs < 2 μg/mL). These analogues were, however, found to be somewhat less active toward *Enterobacteriaceae* (MICs 2–16 μg/mL). For example, the introduction of Cys at P10 (analogue **43**, Figure 6) resulted in MICs of 0.06–0.5 μg/mL against the strains of *P. aeruginosa* tested, while against *E. coli* MICs of 2–4 μg/mL were measured. Interestingly, P10 substitution with Dab (analogue **42**, Figure 6) resulted in reduced antibacterial activity across the board with MICs of 8–32 μg/mL against *P. aeruginosa* and 8–16 μg/mL against *E. coli*. These findings are generally in line with those of the Li group who also recently examined the contribution of the P10 residue to antibacterial activity and toxicity.^88^ A systematic screening of a series of residues at P10 revealed a strong preference for Thr/Ser or hydrophobic residues at this position. While most natural polymyxins contain Thr at P10, there are some variants that instead contain Leu at this position (see Figure 1A above). Notably, the Leu at P10 can be exchanged for a number of other hydrophobic residues, e.g., Ala, Abu, Val, or Nva, without significantly impacting antibacterial activity.^88^ Also of note is the finding that polymyxin analogues bearing P10 substitution do no exhibit appreciably reduced toxicity, either in cell based assays (HK-2 cells)^129^ or in mice (based on observed MTD).^88^

Using a semisynthetic approach, the Huang group also explored the impact of esterifying the hydroxyl groups of Thr at P2 and/or at P10 in polymyxin B with a variety of amino acids. Rather surprisingly, the activities of the esterified analogues thus prepared were reported to be on par with those of the parent polymyxin B species, even when derivatized with bulky amino acids such as Tyr at P2 or acidic residues like Asp at P2 and P10. Notably, analogue **76** (Figure 6, Table 2), featuring a Pro ester at P2 was found to exhibit exceptionally low MIC values, at least one serial dilution better than the parent polymyxin B_2_ while also displaying reduced toxicity toward HK-2 cells. Hydrolysis of these esterified modified polymyxins was also found to occur rapidly upon of incubation at 37 °C in phosphate buffer at pH 7.0, leading the authors to suggest that analogues like **76** may in fact be operating as polymyxin B prodrugs.^129^

### 2.6 Analogues via Novel Semisynthetic Approaches to Access Variation at P6

**Scheme 4:**
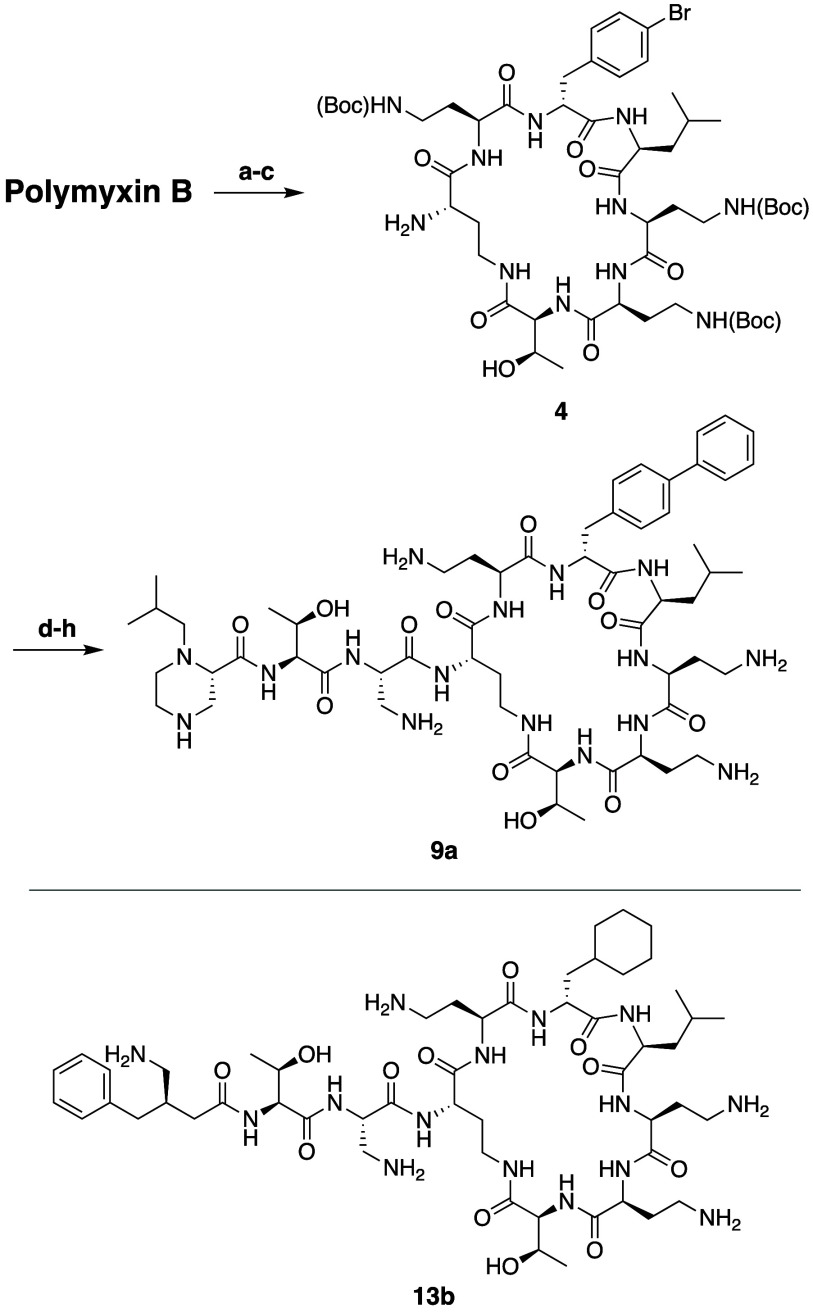
Semisynthesis Route Developed by Brown and Co-workers Providing Access to P6 Modified Polymyxin Analogues Reagents and conditions: (a) *N*-bromosuccinimide, BF_3_·2H_2_O; (b) (Boc)_2_O, Et_3_N, CH_3_CN/H_2_O; (c) Savinase, 1,4-butanediol, phosphate buffer; (d) PhB(OH)_2_, PdCl_2_(PPh_3_)_2_, Na_2_CO_3_, DMF/H_2_O; (e) Cbz-Thr(OtBu)-Dap(Boc)-OH, HATU, DIPEA, DCM; (f) 10% Pd/C, MeOH; (g) Boc-protected building block-COOH, HATU, DIPEA, DCM/DMF; (h) TFA, DCM, followed by purification with RP-HPLC.

A new semisynthetic strategy to further diversity, the polymyxin B scaffold, was described by Brown and colleagues.^130^ This approach provides convenient access to side chain variants extending upon the P6 d-Phe residue found in the natural product. Specifically, by treating polymyxin B with *N*-bromosuccinimide and BF_3_, it was found that the aromatic ring of the d-Phe at P6 can be brominated at the *para*-position. Subsequent Boc protection of the five Dab residues followed by treatment with Savinase (as in previously reported semisynthetic strategies^96^) provides access to the corresponding PMBH[P6-*p*-Br-d-Phe](Boc)_3_ building block **4**, (Scheme 4).^101,130^ Further elaboration was then achieved by introducing different amino acids at P2 and P3, installed as the appropriately protected dipeptides, followed by Suzuki cross-coupling for diversification at P6. Selective deprotection of the *N*-terminus and subsequent acylation with a variety of moieties, followed by global deprotection and purification, provided access to a range of novel semisynthetic polymyxin analogues (Scheme 4). Via this approach analogues such as **9a** were prepared which in some cases were found to exhibit potent antibacterial activity along with slight reductions in cytotoxicity relative to polymyxin B. Also reported by the same authors is the finding that the P6 d-Phe benzene core in polymyxin B can be fully reduced using PtO_2_ under H_2_ atmosphere to give the corresponding d-cyclohexyl alanine analogue.^101,130^ This in turn provided access to analogues such as **13b**, which was also found to exhibit potent antibacterial activity and reduced cytotoxicity.

### 2.7 Analogues Containing Disulfide Linked Peptide Macrocycles

**Figure 7:**
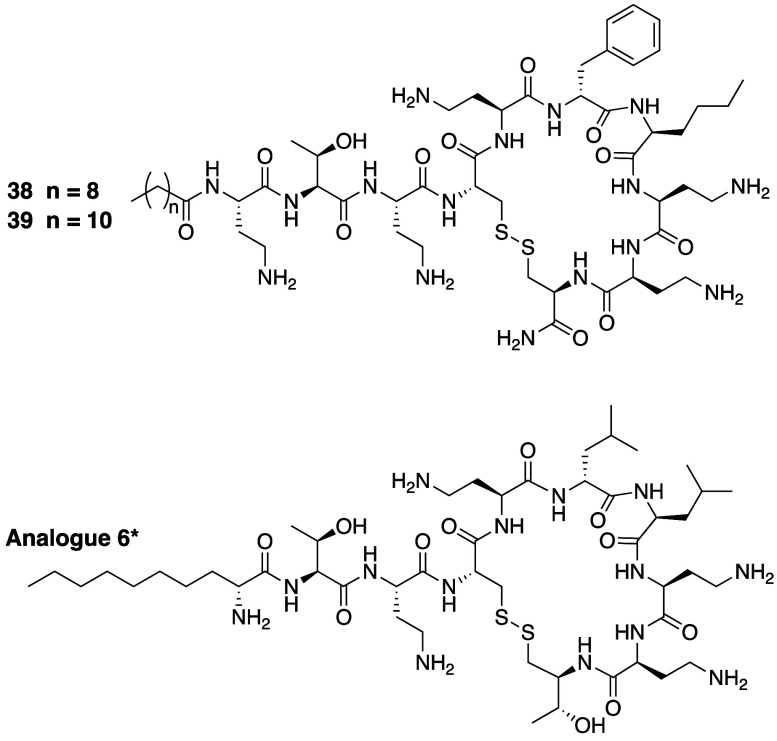
Polymyxin analogues developed by Rabanal and co-workers containing a disulfide-linked macrocycle. *Analogue **6** described in the patent literature.

Rabanal and co-workers reported a unique class of polymyxin analogues, wherein the peptide macrocycle is closed via a disulfide linkage.^131^ This approach was inspired by the somatostatin analogue lanreotide and other clinically used peptide drugs containing disulfide-linked macrocycles. In the approach developed by the Rabanal group, the l-Thr at P10 and Dab at P4 were replaced by l-Cys or d-Cys, facilitating disulfide-mediated ring closure (Figure 7). In addition, the impact of side chain substitutions at P6 and P7 were also explored. Somewhat surprisingly, analogues **38** and **39** were found to have both potent anti-Gram-negative activity, on par with polymyxin B, as well as activity against *S. aureus*, against which polymyxin B is essentially inactive. Analogue **38**, featuring a decanoic acid tail was further evaluated for acute toxicity in CD-1 mice. An LD_50_ value of 283 mg/kg was obtained for **38**, almost 5-fold higher compared to polymyxin B (59.5 mg/kg).^131^ Despite this lower acute toxicity, all disulfide analogues bearing a cysteamide motif at P10 showed nephrotoxicity patterns similar to polymyxin B. Interestingly, data disclosed in a subsequent patent filed by the same group showed that the nephrotoxicity of the disulfide analogues could be reduced by replacing the C-terminal amide moiety with a β-hydroxy group to recapitulate the Thr side chain found at P10 in most polymyxins. Representative analogue **6** contains this modification as well as replacement of the acylated P1 moiety found in natural polymyxins with d-2-amino decanoic acid (Figure 7). The results disclosed in the patent describe these analogues as possessing potent *in vitro* antibacterial activity as well as being less nephrotoxic than polymyxin B *in vivo* based on the number and severity of lesions observed by histopathological inspection of renal samples.^132^ Data demonstrating the *in vivo* efficacy of these analogues in treating bacterial infections has yet to be reported.

### 2.8 Analogues with Reductively Labile *N*-Terminal Lipid Tails

**Figure 8:**
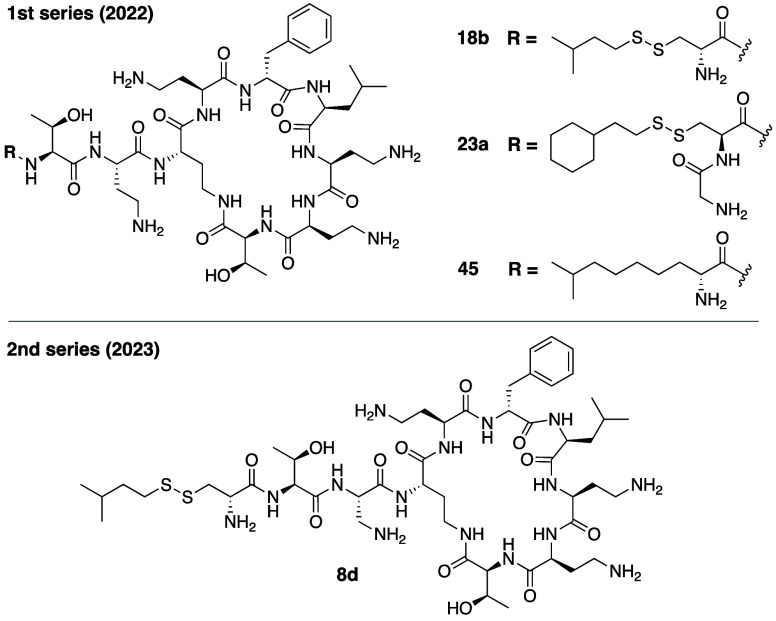
Polymyxin analogues developed by the Martin group containing reductively labile disulfide-linked N-terminal lipids.

With the aim of generating less toxic analogues, our group recently developed a series of novel polymyxin B variants bearing *N*-terminal lipids containing a reductively labile disulfide bond as well as modifications at P1 and P3 (Figure 8).^133,134^ Based on previous studies indicating that the lipid tail contributes significantly to the toxic effects associated with the polymyxins,^35^ we envisioned intracellular cleavage of the disulfide-linked lipid as a means of addressing this toxicity. Given the highly reducing environment found inside kidney cells,^135^ we hypothesized that upon renal uptake, subsequent disulfide reduction could in turn lead to formation of less toxic and/or more rapidly cleared metabolites. In our initial series of analogues, we examined the replacement of l-Dab at P1 with l-Cys or d-Cys, which provided a thiol handle for introducing the disulfide. In doing so, the γ-amino moiety of the Dab side chain normally present at P1 is replaced by an α-amine, bringing it closer to the backbone of the peptide. We therefore also explored the impact of adding a Gly motif to this α-amine to assess the relative importance of the spacing between the primary amine at P1 and the backbone. Using a convenient semisynthetic approach making use of the previously reported PMBN(Boc)_4_,^111^ we prepared a range of analogues by coupling different l-Cys- or d-Cys-based building blocks containing disulfide linked lipids of varying length and branching.

*In vitro* assessment of these disulfide-linked analogues revealed that the structure of the lipid plays an import role in achieving an optimal balance between antibacterial activity and toxicity toward kidney cells (PTECs). To this end, compound **18b** and **23a** were identified as particularly interesting analogues with antibacterial activities comparable to polymyxin B but with reductions in cytotoxicity of 4.7- and 9.5-fold, respectively (Table 2). Also of note is the importance of the stereochemistry of the P1 Cys residue in these analogues. In the case of **18b** where the α-amine moiety is not modified, it is essential to have d-Cys at P1 as the corresponding l-Cys analogue exhibits significantly reduced antibacterial activity. However, when a Gly residue is coupled to the α-amine moiety of the Cys residue at P1, the stereochemical requirement is reversed. In this case, analogue **23a**, containing l-Cys at P1, was found to be more potent and less nephrotoxic than the analogue containing d-Cys at the same position.

To establish the impact of the disulfide motif on the reduced toxicity observed for these analogues we synthesized and tested **45**, the all-carbon analogue of **18b**. While **45** was found to exhibit antibacterial activity on par with **18b**, it was also found to be 2.4 times more toxic toward PTECs (CC_50_ of 82 μM for **45** vs 192 μM for **18b**). This would suggest that reductively labile disulfide linkage in the lipid tail does have a positive effect in reducing toxicity. We also tested the hypothesis that the disulfide linkage incorporated in these polymyxin analogues could be selectively cleaved under different reducing conditions. To this end compounds **18b** and **23a** were incubated with high (5 mM) and low (5 μM) glutathione concentrations meant to mimic the glutathione concentrations found inside proximal tubular cells and in blood.^135,136^ While **18b** and **23a** were both rapidly degraded under conditions mimicking the higher intracellular concentration of glutathione (undetectable after 1 h), in the low glutathione conditions the compounds were found to be 70–80% intact after 24 h incubation at 37 °C. These findings suggest that the inclusion of disulfide-linked lipids may indeed provide a means to tune the metabolic stability of polymyxins in pursuit of analogues with reduced toxicity. Building from these findings we also recently reported a second generation of disulfide linked analogues based on **18d** wherein the P3 residue was varied.^134^ This led to the identification of analogue **8d**, bearing l-Dap at P3, as an improved variant with potent antibacterial activity and even lower cytotoxicity toward PTECs (Table 2).

### 2.9 Analogues Bearing *N*-Terminal Lipids Containing Thioether and Ester Linkages

**Figure 9:**
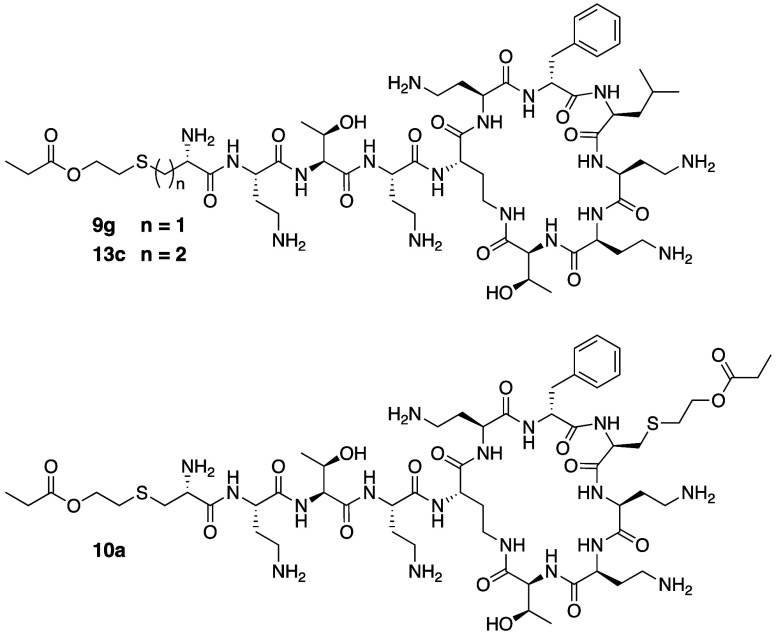
Polymyxin analogues reported by Harris and Brimble bearing N-terminal lipids containing thioether and ester linkages.

Another class of polymyxin analogues bearing modified *N*-terminal lipid moieties was recently reported by the group of Harris and Brimble wherein structural variation was introduced by employing thioether linked modifications.^137^ In these analogues, a number of *S*-alkylated Cys-derived building blocks were coupled to the *N*-terminus of the polymyxin decapeptide (Figure 9). Among the analogues thus prepared, those containing both the thioether and a hydrolytically labile ester linkage in the *N*-terminal lipid, similar to the polymyxins developed by MicuRx, were found to be most promising. Specifically, analogues **9g** and **13c** (Figure 9, Table 2) showed activity comparable to polymyxin B against a panel of Gram-negative bacteria while exhibiting reduced toxicity in an in vitro kidney organoid model. Notably, the use of organoid-based models presents a new approach to assessing polymyxin toxicity and may be of improved predictive value in recapitulating some of the spatial features found in renal tissue.

The same group also explored the effect of introducing additional *S*-linked lipid moieties at P6 and P7 in combination with those incorporated at the *N*-terminus. Representative analogue **10a** includes a P7 substitution with a sulfur-derivatized Cys, alkylated with the same ester-containing lipid appended to the *N*-terminus (Figure 9, Table 2). While this compound was found to perform reasonably well against *Enterobacteriaceae* (MICs 2–4 μg/mL), it showed reduced activity against *P. aeruginosa* and *A. baumannii*. These observations, in combination with the finding that **10a** is not significantly less toxic than analogue **9g**, indicate that *S*-linked substitutions at P6 and P7 offer less benefit compared to the effects associated with *N*-terminal modification. Further assessment of **9g** revealed that while it was relatively stable in organoid medium (ca. 60% remaining after 24 h), in plasma it was rapidly degraded (9% remaining after 4 h with near complete degradation within 24 h). Whether this strategy can lead to optimized polymyxins with a desired activity-toxicity-stability balance *in vivo* remains an open question and will likely require further fine-tuning of the ester linkage in the *N*-terminal lipid moiety as for the MicuRx analogues.^110^

## 3 Polymyxin Analogues with Only Antibacterial Activity Reported

### 3.1 Analogues with Increased Hydrophobicity

**Figure 10:**
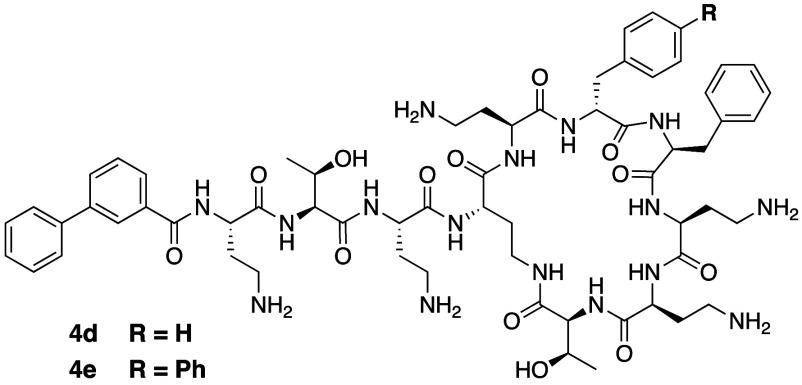
Polymyxin analogues prepared by Ichikawa and co-workers to investigate the impact of introducing alternative hydrophobic groups at the N-terminus as well as P6 and P7.

Ichikawa and co-workers reported a series of polymyxin derivatives focused on revealing the effect of increasing hydrophobicity at both the *N*-terminus as well as at P6 and P7.^138^ Using a total synthesis approach a number of novel analogues were prepared containing branched aliphatic or biaryl moieties at these positions. Antibacterial activity was found to be abolished upon incorporation of long linear (C_18_) or branched (bis-C_8_) lipids at the *N*-terminus. By comparison, some activity was retained upon incorporation of biaryl lipids at the *N*-terminus (MIC against *E. coli* of 8 μg/mL), in line with previous reports.^125^ However, the introduction of biaryl substituents at P6 or P7 in combination with the incorporation of similar motifs at the *N*-terminus led to a significant loss of activity (compounds **4d** and **4e**, Figure 10, Table 2). Given that an assessment of the toxicity of these analogues was not reported it is difficult to assess their actual potential.

### 3.2 Analogues with *N*-Terminal Bis-Lipidation

**Figure 11:**
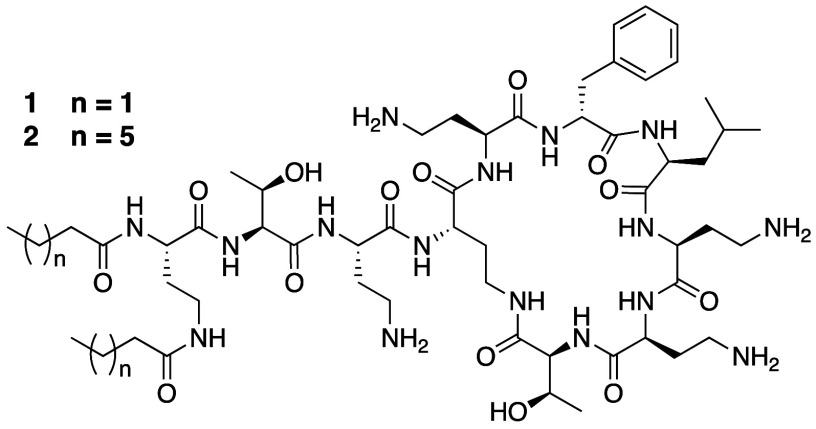
Bis-lipidated polymyxin analogues prepared by Schweizer’s group.

Another series of polymyxins with increased *N*-terminal hydrophobicity were reported by the group of Schweizer wherein both the *N*-terminal α-amine and the side chain amine of the P1 Dab residue were acylated with fatty acids of varying hydrophobicity.^139^ When tested against a panel of Gram-negative pathogens, the bis-C_4_ lipidated polymyxin B analogue **1** (Figure 11, Table 2) was found to have little-to-no activity (MIC ≥ 64 μg/mL on all strains) indicating that two *N*-terminal C_4_ lipids do not recapitulate the effect of a single C_8_ lipid. In keeping with this finding, bis-C_8_ lipidated analogue **2** showed improved antibacterial potency, especially against the strains of *P. aeruginosa* tested (MICs 2–8 μg/mL). This enhanced anti-*P. aeruginosa* activity was also observed for analogues containing bis-biphenyl or bis-adamantyl motifs at the same positions. In search of an explanation for the observed specificity of these analogues against *P. aeruginosa*, the authors identified a potential role for reduced bacterial efflux. This was based on the finding that the MIC of **1** was significantly reduced (from 128 μg/mL to 2–4 μg/mL) on a *P. aeruginosa* strain that lacks the MexAB-oprM efflux pump. In contrast, the inherently more potent analogue **2** did not perform better on the same efflux mutant strain, leading to the conclusion that bis-lipidation with the appropriate length of lipid may serve to circumvent efflux. It is also noteworthy that these bis-lipidated polymyxins were in general found to be slightly more hemolytic than the natural polymyxins.^139^ In order to assess their potential more fully, further studies in relevant models of kidney injury are needed.

## Conclusions and Outlook

The polymyxins remain a clinically important class of lipopeptide antibiotics given their potent activity against problematic Gram-negative bacteria. This activity is driven by their ability to target lipids that are abundant and exclusive to the Gram-negative OM, most notably the LPS anchor Lipid A.^7−9^ To date, a range of polymyxin variants from natural sources have been identified and, using the tools of modern biotechnology and chemistry, are accessible via fermentation or total synthesis.

At present, polymyxin B and colistin are the only polymyxins used clinically. However, their clinical application is limited due to their notorious dose-limiting toxicity, most typically nephrotoxicity.^36−41^ These toxic effects are primarily attributed to excessive uptake of the drug in proximal tubule epithelial cells, via the endocytic receptors megalin, the carnitine/organic cation transporter 2 (OCTN2), and oligopeptide transporter 2 (PEPT2).^46,49−53^ Potassium channels have also recently been implicated in the nephrotoxic effects of the polymyxins.^54^ Ultimately, excessive polymyxin accumulation throughout kidney cells results in oxidative stress, in turn leading to apoptosis.^62,65^

Given the potent antibacterial activity of the polymyxins and their increasing use in treating infections due to multidrug resistant bacteria, a strong case can be made for developing next-generation analogues with improved therapeutic indices. In recent years three new clinical candidates have been developed each with a different approach for reducing the toxicity of polymyxins. Spero pharmaceuticals’ **SPR206** (Scheme 1) is a P1 truncated analogue that contains an optimized *N*-terminal acyl group and a Dap for Dab P3 substitution.^100^ By comparison, Shiongi’s **QPEX9003** is a full length polymyxin E analogue bearing an *N*-terminal aromatic acyl group, the same Dap for Dab substitution at P3, and a slightly less hydrophobic side chain at P7.^55^ Distinct from these is MicuRx’s metabolically labile polymyxin B analogue **MRX-8** which aims to employ a different strategy to address polymyxin’s toxicity, namely *in vivo* detoxification by enzymatic hydrolysis.^110^

Benefiting from decades of investigation and effort on the part of many groups, novel polymyxin analogues can today be generated by a range of robust and reliable strategies. Depending on the desired region of structural variation, robust semisynthesis and total synthesis methods offer a wide range of possibilities. Notably, the optimized and highly automated SPPS approaches recently developed provide convenient access to new polymyxins in quantities suitable for explorative studies. Whether such SPPS approaches can be viable for the much larger-scale synthesis of a clinically used next-generation polymyxin remains to be seen. With respect to the individual amino acids found in the natural polymyxins, the Dab residues at P1 or P3 are particularly amenable to modification leading to analogues with enhanced activity and/or reduced toxicity.^34,117^ By comparison, the Dab moieties at P5 and P9 are much less tolerant of substitution, leaving the P6/P7/P8 region of the polymyxin macrocycle as targets for structural change. Generally, variations to P6/P7/P8 are tolerated so long as the hydrophobic character of this region is maintained.^124^

While significant gains have been made in generating SAR insights related to the antibacterial potency of polymyxin analogues, predicting their *in vivo* toxicity remains a challenging task. Whereas cellular uptake is typically accounted for in cell culture assays, long-term accumulation effects are not observable given the shorter time course of such experiments. Despite this limitation, screens using HK-2 cells or PTECs are still frequently employed in the initial estimation of polymyxin toxicity.^34,100,110,121,133^ As an alternative, acute toxicity in mice can be used as a proxy for toxicity in humans, recapitulating not only the nephrotoxicity but general toxicity as well.^55,88,140^ However, results from animal-based assays do not offer guaranteed transferability to human medicine.^34^ In this regard, toxicity assessment based on the use of organoids derived from human cells may offer an additional option for assessing polymyxin toxicity, underscoring the value in further establishing such models.^137^

It is clear that the polymyxins will continue to be of clinical relevance in the coming decades as will interest in the pursuit of new analogues with improved safety profiles. The steadily growing wave of resistance among Gram-negative pathogens, along with the lack of new LPS/lipid A targeting modalities in the pipeline, has led to a renewed appreciation for the polymyxins as critical last resort antibiotics. In this light, the successful development of next-generation polymyxins with potent antibacterial activity and reduced toxicity will critically depend on sustained efforts firmly grounded in medicinal chemistry.

## Abbreviations

AA: amino acid
Abu: amino butyric acid
AFM: atomic force microscopy
AMR: antimicrobial resistance
ATCC: American Type Culture Collections
BF_3_: boron trifluoride
BGC: biosynthetic gene cluster
CC_50_: concentration with 50% cytotoxicity
CFU: colony forming units
CMS: colistin methanesulfonate
CTC: chloro trityl chloride
Dab: 2:4-diamino butyric acid
Dap: 2,3-diamino propionic acid
DIPEA: *N*,*N*′-diisopropylethylamine
DPPA: diphenylphosphoryl azide
(ES)KAPE: (*Enterococcus faecium*, *Staphylococcus aureus*) *Klebsiella pneumoniae*, *Acinetobacter baumannii*, *Pseudomonas aeruginosa*, *Enterobacter* spp
FICI: fractional inhibitory concentration index
GGT: γ-glutaryl transferase
HABP: hospital-acquired bacterial pneumoniae
IM: inner membrane/cytoplasmic membrane
ITC: isothermal titration calorimetry
LDH: lactate dehydrogenase
LPS: lipopolysaccharide
MDR: multidrug resistant
MIC: minimum inhibitory concentration
MTD: maximum tolerated dose
NCTC: National Collection of Type Cultures
NDM: New Delhi β-metallo lactamase
Nle: norleucine
Nva: norvaline
NRPS: nonribosomal peptide synthesis
OM: outer membrane
OMV: outer membrane vesicle
PK/PD: pharmacokinetic(s)/pharmacodynamic(s)
PMBH: polymyxin B heptapeptide
PMBN: polymyxin B nonapeptide
PmxB: polymyxin B
PT: proximal tubule
PTEC: proximal tubule epithelial cell
ROS: reactive oxygen species
SPPS: solid phase peptide synthesis
TNF: tumor necrosis factor
VABP: ventilator associated bacterial pneumoniae
